# Cell death signaling and immune regulation: new perspectives on targeted therapy for sepsis

**DOI:** 10.1186/s11658-025-00784-w

**Published:** 2025-08-15

**Authors:** Huang Wu, Jiale Cui, Jie Huang, Yuqi Feng, Jiaxin Zhao, Yalin Zhu, Xiaoming Deng, Xinyu Li, Wangzheqi Zhang, Changli Wang

**Affiliations:** 1 Faculty of Anesthesiology, Changhai Hospital, Naval Medical University, Shanghai, 200433 China; 2 Basic Medical University, Naval Medical University, Shanghai, 200433 China; 3 School of Anesthesiology, Naval Medical University, 168 Changhai Road, Shanghai, 200433 China; 4 Department of Anesthesiology, Naval Hospital of Eastern Theater, Zhoushan, 316004 China; 5 Department of Burn Surgery, Changhai Hospital, Navy Medical University, Shanghai, 200433 China

**Keywords:** Cell death, Sepsis, Inflammation, Immunosuppresion, Immune regulation, Signaling

## Abstract

Cell death is essential for the preservation of tissue homeostasis, regulating inflammatory responses, and shaping immune status. The mechanism of cell death includes apoptosis, pyroptosis, necroptosis, ferroptosis and autophagy. The onset, progression, and unfavorable prognosis of sepsis are closely associated with these pathways. Here, the mechanisms associated with these five major cell death pathways in sepsis are reviewed, emphasizing two core aspects of the condition: excessive inflammation and immune suppression. These pathways play a fundamental role in modulating these characteristics and offer novel therapeutic prospects. The study provides valuable insights and detailed analyses, making a significant contribution to ongoing research in this domain. The interconnected nature of cell death is highlighted, not only by examining the distinct roles of individual pathways but also by exploring the interactions between different pathways and the crosstalk among key signaling molecules or pathways, including the caspase family, gasdermin family, and NF-κB pathway. Further research should continue to investigate well-established cell death mechanisms while also identifying previously unknown pathways. Therapeutic strategies targeting cell death pathways hold broad application potential. However, during the transition from preclinical research to clinical application, several challenges remain, including limitations of experimental models, as well as the safety and efficacy of treatments. Additionally, the development of personalized treatment approaches tailored to the unique immune profiles of patients is crucial for advancing precision medicine. In conclusion, the present review offers an extensive analysis of the diverse roles of cell death in sepsis, with novel insights into disease mechanisms and guiding therapeutic developments.

## Introduction

Sepsis contributes significantly to mortality and critical illness globally. Its pathogenesis is characterized by dysfunction in multiple organs caused by atypical host responses to infection [1]. Data from high-income countries indicate that each year, more than 31 million sepsis cases are reported in hospital settings, with 19.4 million progressing to severe disease and a further 5 million resulting in death [2]. In middle- and low-income countries (taking data from Blantyre, Malawi, from 2013 to 2016 as an example) the overall population incidence rates of sepsis and severe sepsis were 1772 cases per 100,000 individuals and 303 cases per 100,000 individuals, respectively [3]. Due to the extensive damage inflicted by sepsis on the body and its substantial impact on health worldwide, extensive research has been conducted to elucidate its pathophysiological mechanisms and identify effective therapeutic interventions.

Sepsis is primarily an inflammatory condition resulting from dysregulated innate immune responses. Its pathogenesis involves the recognition of microbial components or endogenous signaling molecules by specific cell surface receptors and the complement system. This recognition initiates downstream signaling cascades, leading to alterations in gene expression and subsequent inflammatory responses [2]. As the condition advances, its primary manifestations shift from excessive inflammation to immune suppression, characterized by decreased immunocyte numbers, functional impairment, and suppressed cytokine production. This condition has been identified as Persistent Inflammation, Immunosuppression, and Catabolism Syndrome (PICS) [2, 4, 5]. During the pathophysiological process of sepsis, cell death is one of the key mechanisms leading to the inflammatory response and immune suppression [6]. Compared to non-septic individuals, those with sepsis exhibit significantly increased lymphocyte apoptosis [7]. Pyroptosis is linked to inflammatory responses and immunological suppression and is markedly elevated in sepsis patients [8]. In addition to pyroptosis, cell death mediated by necroptosis, ferroptosis, and autophagy significantly influences inflammation and immune suppression during sepsis [9–11]. The pathophysiological processes of sepsis are further intensified by the interactions among these cell death pathways, underscoring the complexity and heterogeneity of disease progression.

Elucidating the contributions of cell death pathways to sepsis pathophysiology is essential for achieving precise disease control and effective therapeutic interventions [12]. This review explores the specific roles of five major and well-characterized forms of cell death, namely, apoptosis, pyroptosis, necroptosis, ferroptosis, and autophagy, in sepsis progression. The influence of these cell death pathways on two fundamental aspects of sepsis, excessive inflammation and immune suppression, is systematically analyzed [2]. Furthermore, the interconnections and interactions among these five cell death pathways are examined to uncover their synergistic or antagonistic effects in sepsis development and to identify potential molecular targets. Modulation of these cell death pathways may provide an effective strategy for controlling excessive inflammation and precisely regulating immune function in sepsis. This study seeks to comprehensively analyze possible correlations and interactions among different cell death pathways, explore novel targeted therapeutic strategies the challenges and prospects of clinical translation, and optimize clinical treatment approaches for sepsis. This effort is expected to contribute to improving sepsis cure rates with long-term benefits for affected patients.

## Cell death pathways in sepsis

**Fig. 1:**
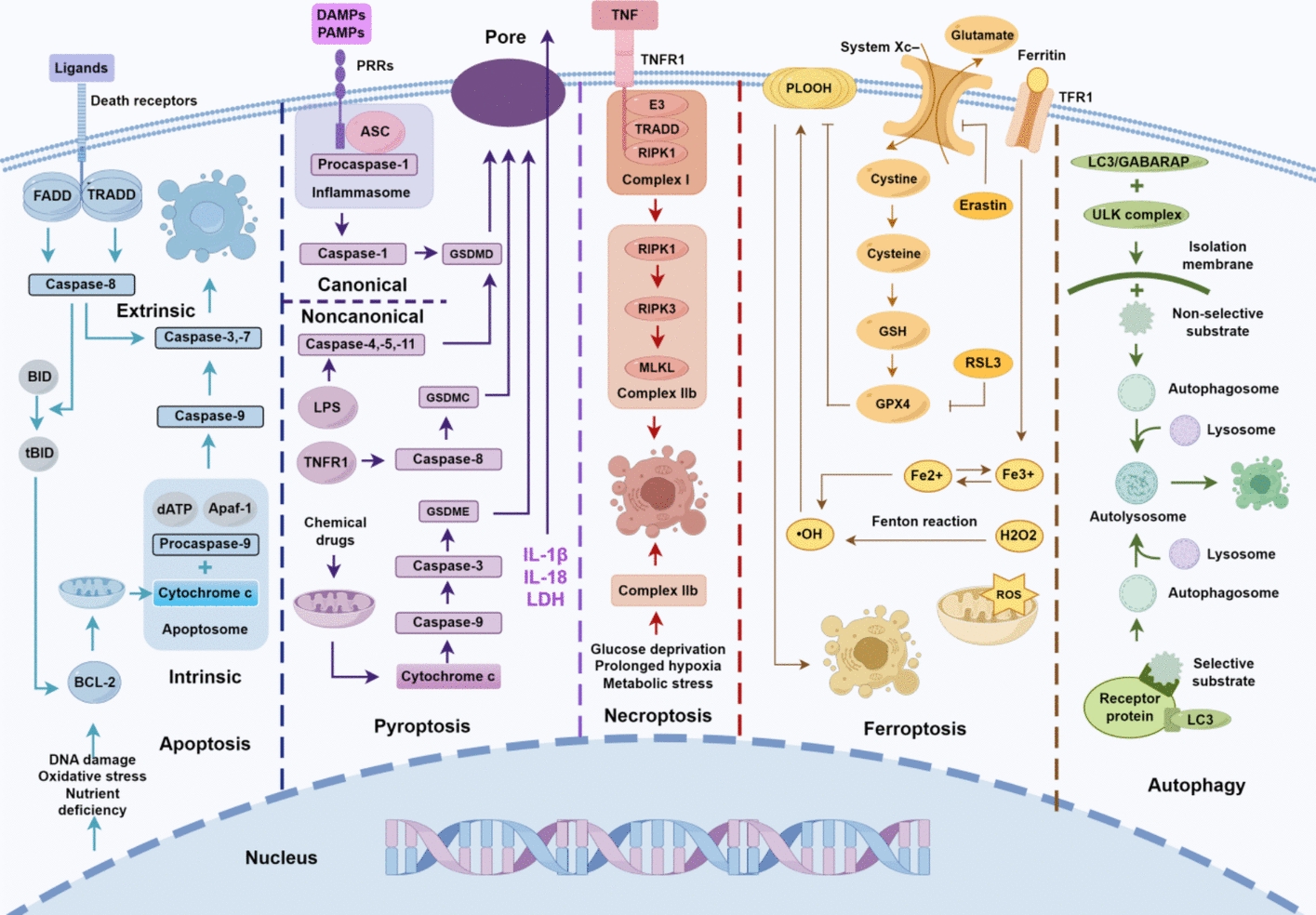
Five major cell death pathways. ① The extrinsic apoptotic pathway is initiated when ligands bind to death receptors, activating caspase-8 through FADD or TRADD, which in turn activates caspase-3 and -7 to execute apoptosis. The intrinsic pathway begins with BH3-only BCL-2 proteins, which modulate the release of mitochondrial cytochrome c, form the apoptosome, and activate caspase-9, followed by caspase-3 and -7. Both pathways can converge through caspase-8-mediated cleavage of Bid. ② The canonical pyroptosis pathway begins with the recognition of DAMPs and PAMPs by PRRs, which form inflammasomes that activate caspase-1. Caspase-1 cleaves and oligomerizes GSDMD to create pores in the cell membrane. The non-canonical pyroptosis pathway is triggered by bacterial LPS, TNFR1, and certain chemicals, leading to pore formation via the activation of GSDMC, GSDMD, and GSDME. ③ In the necroptosis pathway, TNFR1 activation by TNF-α recruits TRADD and RIPK1 to form complex I, which phosphorylates RIPK1, activating RIPK3 and MLKL. These proteins form complex IIb, promoting necroptosis, particularly under damaging conditions. ④ Ferroptosis is characterized by the accumulation of lipid peroxides on cell membranes, disrupting ion balance and causing cell swelling. Key molecules involved include GPX4 (with GSH) and the system xc^–^. Inhibition of GPX4 by RSL3 increases PLOOH levels, leading to ferroptosis, while erastin inhibits system xc^–^, reducing GSH synthesis and also inducing ferroptosis. ⑤ Autophagy is classified into selective and non-selective types. Under nutrient deprivation, the ULK complex initiates the formation of an isolation membrane with LC3/GABARAP proteins, which expands to engulf non-selective substrates, creating autophagosomes that fuse with lysosomes to form autolysosomes. Selective autophagy specifically targets substrates for autophagosome formation, utilizing receptor proteins that link selective substrates to LC3 via ubiquitin-binding and LIR domains. This interaction aids in the formation of the isolation membrane, which matures into autophagosomes and autolysosomes. (By Figdraw)

Cell death is fundamental to normal tissue maturation and the maintenance of systemic homeostasis. By removing excess cells, it ensures the proper morphology and function of tissues and organs [13]. The elimination of abnormal cells, including damaged, tumor, and autoreactive immune cells, is essential for sustaining internal equilibrium and systemic stability [14, 15]. However, in sepsis, excessive death of immune cells can have significant adverse effects on defense against pathogens, tumors, and harmful substances. Conversely, inadequate immune cell death may result in an overactive immune response and uncontrolled inflammation, both of which can severely compromise health [2]. In the context of sepsis, a systematic evaluation is conducted to analyze the mechanisms driving five major cell death forms, namely, apoptosis, pyroptosis, necroptosis, ferroptosis, and autophagy. By integrating the mechanisms associated with these pathways, this review seeks to extend knowledge regarding the complex mechanisms underlying sepsis and to support the development of new methods of treatment. Figure 1 details the characteristics of the pathways.

### Sepsis-associated apoptosis

Apoptosis, classified as a programmed cell death mechanism, is essential for regulating inflammatory responses, cell proliferation, and tissue regeneration [16]. Apoptotic cells exhibit distinct changes in morphology, including DNA fragmentation, the development of cytoplasmic vacuoles, and membrane blebbing, while releasing specific metabolites that influence the surrounding microenvironment [17]. Apoptotic processes preserve the integrity of plasma membranes, thereby restricting the release of intracellular material [14]. As a key regulatory mechanism, apoptosis is signficantly linked to both developmental and repair processes, and its disruption can result in pathological changes [18]. Therefore, precise regulation of apoptosis is critical for maintaining physiological balance and addressing pathological challenges.

Apoptosis is primarily categorized into intrinsic and extrinsic pathways based on whether it is mediated by receptors in the plasma membrane. The former is typically activated when responding to harmful conditions, including DNA damage, oxidative stress, and nutrient deprivation [19]. During this process, the levels of anti-apoptotic members of the Bcl-2 family are decreased, while those of pro-apoptotic members are raised, resulting in cytochrome C release from the mitochondria [20]. This cytochrome C participates in the formation of apoptosomes, resulting in caspase-9 activation and initiating a cascade activating caspases 3 and 7, thereby inducing apoptosis [21, 22]. The extrinsic pathway is triggered when death receptors, including Fas, TNFR1, and TRAILR1/2, engage with their ligands, including FasL, TNF, and TRAIL. Upon ligand binding, Fas and TRAILR1/2 recruit Fas-associated protein with death domain (FADD) to activate caspase-8. At the same time, TNFR1 interacts with TNF receptor type 1-associated death domain protein (TRADD) to activate caspase-8. Ultimately, apoptosis is induced by activating caspases-3 and -7 [23].

Apoptosis can control the numbers of immune cells during sepsis and is extensively observed across multiple organs [24]. It has been found that under septic conditions, the proportion of neutrophils displaying apoptotic morphological changes decreases by about 40% after 24 h of in vitro culture compared to resting neutrophils. This suggests that the establishment of a neutrophil-enriched environment hostile to apoptosis may promote systemic inflammation and organ damage in sepsis [25, 26]. In clinical sepsis patients, a negative correlation has been observed between neutrophil apoptosis and sepsis severity, indicating that reduced neutrophil apoptosis may significantly increase the damage to tissues and organs [26, 27]. Beyond neutrophils, lymphocyte apoptosis, particularly involving B and CD4^+^ T cells, is considered a key factor in sepsis-induced immune suppression [28]. Apoptotic lymphocytes exhibit anti-inflammatory properties and contribute to immune tolerance [29, 30]. For instance, the production of pro-inflammatory cytokines in lymphocyte is reduced when apoptotic cells are phagocytosed, accompanied by increases in anti-inflammatory factors, a pattern consistent with observations in sepsis patients [31, 32]. Additionally, an elevated proportion of regulatory T cells (Tregs) is observed in cases of septic shock. These Tregs induce apoptosis of CD4^+^ effector T cells via the transforming growth factor β1 (TGF-β1) axis, thereby reinforcing anti-inflammatory activities through a negative feedback loop [33, 34]. The apoptosis of these lymphocytes is associated with more pronounced immune suppression, heightened susceptibility to infections, and increased mortality risk [35–37].

In summary, activation of caspases by the intrinsic and extrinsic pathways results in apoptosis, with consequences for various physiological and pathological functions. During sepsis, the promotion of apoptosis in immune and parenchymal cells disrupts the equilibrium between pro- and anti-inflammatory processes, adversely affecting immune barriers, which significantly influence sepsis prognosis. Targeting apoptosis-related molecules may aid in controlling the progression of sepsis. However, selective inhibition of apoptosis must be approached with caution to prevent excessive suppression or unintended inhibition of certain immune cell apoptosis, which could exacerbate inflammatory responses. Therefore, an in-depth understanding of these interconnections and the discovery of key molecules involved could facilitate more effective regulation and treatment strategies for sepsis, ultimately improving patient survival outcomes.

### Sepsis-associated pyroptosis

Pyroptosis is a non-apoptotic process closely associated with inflammatory responses [38]. The inflammasome serves as a central component in the pyroptosis process, activating downstream caspases, which subsequently induce pore formation in cell membranes by gasdermin proteins. This pore formation compromises membrane integrity, resulting in leakage of intracellular contents and inducing inflammation [14, 39, 40].

Pyroptosis is regulated by both canonical and non-canonical pathways. The former is associated with the detection of damage-associated molecular patterns (DAMPs) on damaged or cancerous cells and pathogen-associated molecular patterns (PAMPs) by pattern recognition receptors (PRRs) [41]. PRRs interact with apoptosis-associated speck-like protein (ASC) and procaspase-1, forming inflammasomes and activating caspase-1, which then induces the release of the inflammatory IL-1β and IL-18 cytokines and cleavage of gasdermin D (GSDMD). The cleaved GSDMD N-terminal peptide undergoes oligomerization, inducing membrane pore formation. These pores transfer IL-1β, IL-18, and other molecules, such as lactate dehydrogenase (LDH), from the cell to the exterior environment, enhancing inflammation [42–44]. Multiple mechanisms contribute to non-canonical pyroptosis mechanisms. For example, the CARD domain of caspases-4, -5, or -11 interacts directly with bacterial lipopolysaccharide (LPS), leading to GSDMD-mediated pore formation [41, 45]. In addition, activation of caspase-8 by the death receptor-6 (DR6) axis can lead to pore formation via gasdermin C (GSDMC) [46]. Upon exposure to specific chemical agents, mitochondria release cytochrome c, leading to first caspase-9 and then caspase-3 activation, and leading to pore formation through gasdermin E (GSDME) [14, 39, 47].

Pyroptosis has protective functions by depriving intracellular bacteria of the conditions necessary for replication while activating effector cells to clear pathogens [48]. However, it can also contribute to increased mortality through the canonical pathway by promoting IL-1β release [49]. Emerging research suggests that sepsis progression may be influenced by neutrophil pyroptosis [50]. Neutrophils exhibit resistance to canonical caspase-1-mediated pyroptosis, allowing for prolonged survival. This enables sustained IL-1β production at infection sites without compromising antimicrobial functions [51, 52]. Nevertheless, during sepsis, non-canonical inflammasomes can be activated in LPS-transfected neutrophils, inducing pyroptosis [52]. In murine sepsis models, reducing neutrophil extracellular traps (NETs) can improve organ function and survival, with caspase-11 playing a role in NET formation. Caspase-11 knockout mice exhibit decreased NET release from neutrophils and are protected from CLP-induced sepsis [53]. Therefore, neutrophil pyroptosis exacerbates organ damage in sepsis through NET release [26]. According to the Gene Expression Omnibus database, the level of miR-21 in the peripheral blood of patients with septic shock is elevated. The absence of miR-21 can inhibit pyroptosis by suppressing the expression of NLRP3, ASC, and caspase-1 in myeloid cells, as well as the formation of the inflammasome [54]. Beyond the immune system, pyroptosis in other systems is also involved in sepsis pathophysiology [8]. In the coagulation system, tissue factors within the extrinsic coagulation pathway can be activated by bacterial endotoxins, initiating the coagulation cascade through "gasdermin channels" formed by the activation of caspase-11 [55]. Furthermore, macrophage pyroptosis has been shown to be inhibited by platelet endothelial cell adhesion molecule-1, thereby preventing sepsis-related disseminated intravascular coagulation (DIC) and aiding in the restoration of vascular permeability barriers following inflammatory stimulation [56]. The knockout of caspase-11 in sepsis-related acute lung injury (ALI) prevents the aggregation of neutrophils induced by endotoxemia, as well as reducing lung edema and mortality [57]. In sepsis-induced cardiac dysfunction, upon LPS stimulation, IRF3 positively modulates the levels of NOD-like receptor family pyrin domain-containing 3 (NLRP3), increasing cardiomyocyte pyroptosis and worsening myocardial injury [58]. The blood–brain barrier (BBB) serves as a crucial physiological barrier of the central nervous system, playing an essential role in defending against infections and the invasion of harmful substances [59]. LPS is capable of activating the pyroptosis pathway mediated by caspase-11 in brain endothelial cells (bECs). In this process, pore formation induced by GSDMD is the core mechanism that triggers BBB disruption associated with sepsis. Further research has demonstrated that the expression of a specific GSDMD-neutralizing nanobody in brain endothelial cells can effectively block this destructive process, thereby offering a potential therapeutic strategy to preserve the integrity of the BBB [60].

In summary, inflammatory responses are strongly associated with pyroptosis primarily through gasdermin regulation by caspases. This activation induces pore formation in membranes with subsequent extrusion of intracellular material, influencing immune responses and inflammatory processes [61]. In the context of sepsis, pyroptosis has dual effects: it enhances microbial clearance and aids in preventing sepsis-associated DIC, while excessive pyroptosis exacerbates organ injury and is positively correlated with cardiac and pulmonary dysfunction [50]. Targeting key molecules such as caspases and gasdermins to modulate pyroptosis intensity has gained recognition as a promising treatment for sepsis and its accompanying organ injuries [62]. Advancing this knowledge is expected to improve sepsis and septic shock treatment, reduce sepsis-related mortality, and enhance patient quality of life.

### Sepsis-associated necroptosis

The host immune response to microbial infections relies on necroptosis, a regulated necrotic pathway characterized by both passive and active pro-inflammatory roles [47]. Activation of necroptosis initiates a cellular self-destruction program, causing the passive secretion of cytokines, DAMPs, and PAMPs into the extracellular environment [63]. These released molecules facilitate the recruitment of immune cells to the infection site, where they are recognized, thereby triggering antimicrobial responses and promoting tissue repair [64].

TNFR1 is activated to recruit and synthesize complex I, which contains TNFR1 related death domain protein (TRADD), inhibitor of apoptosis 1 (cIAP1), cIAP2, etc. In the TNFR1 signaling complex I, RIPK1 is phosphorylated. It is polyubiquitinated by molecules such as cIAP1 and cIAP2, which in turn triggers the activation of pro-inflammatory and pro-survival transcription factors nuclear factor-κB (NF-κB) and MAPK. Meanwhile, phosphorylation and ubiquitination events of RIPK1 also inhibit its dissociation from complex I [65]. Phosphorylated RIPK1 then binds and activates RIPK3 through its RIP homotypic interaction motif (RHIM), subsequently phosphorylating mixed lineage kinase domain-like (MLKL) to mediate necroptosis [65–67]. Besides, through RIPK1-independent pathway, activated Z-DNA-binding protein 1 (ZBP1) and TIR domain-containing adapter-inducing interferon-β(TRIF) assemble with RIPK3, forming a necrosome and thereby inducing necroptosis [47, 65, 68, 69].

Necroptosis is an essential component of inflammation-related cell death. In the immune system, dendritic cells (DCs) are a pivotal factor contributing to immune dysregulation in sepsis [70]. Peripheral blood samples from non-surviving septic shock patients showed that monocyte-derived DCs (MDDCs) undergo necroptosis, leading to functional impairment and reduced cell numbers. This process may contribute to the excessive inflammation and immune suppression observed in severe sepsis [71–75]. A study analyzing inpatients demonstrated that patients with severe sepsis and septic shock had markedly higher RIPK3 levels than those with general sepsis, and that RIPK3 levels were positively correlated with SOFA scores and PCT levels [76]. Necroptosis is also a significant factor in peripheral organ damage during sepsis. Blocking of necroptosis can mitigate lung, kidney, and liver injuries in sepsis [77–79]. Alveolar macrophages (AMs), the major immune cell type in the lung, are closely involved in ALI progression following both infectious and non-infectious stimuli by producing and releasing various inflammatory mediators [80–83]. In septic shock animal models, knockout of RIPK3, a key necroptosis-associated molecule, reduces circulating cell death markers and enhances AM survival, thereby mitigating sepsis-induced lung injury [84, 85]. These results indicate that necroptosis of AMs is closely linked with sepsis progression and complications. A time-dependent rise in RIPK3 and p-MLKL levels has been reported in HK-2 proximal tubule cells following LPS stimulation. Similarly, raised p-RIPK3, RIPK3, and MLKL levels have been found in renal tissues of mouse models of sepsis resulting from cecal ligation and puncture (CLP). These findings demonstrate that necroptosis of renal tubular epithelial cells contributes to SA-AKI pathogenesis and progression [86–88].

Necroptosis, controlled by the RIPK1-RIPK3-MLKL axis, and its related disruption of cell membranes and consequent release of intracellular material contribute to inflammation [89]. Necroptosis is strongly related to damage to major organs, such as the liver, kidneys, and lungs. Targeting necroptosis-related molecules has emerged as a vital strategy for reducing sepsis complications and enhancing patient survival. MLKL, a key necroptosis molecule, can be antagonized via small molecules binding to its nucleotide-binding site [90, 91]. Additionally, necrostatin-1 (Nec-1), a RIPK1 inhibitor, effectively suppresses necroptosis, showing therapeutic promise in fatal systemic inflammatory response syndrome (SIRS) and sepsis [84].

### Sepsis-associated ferroptosis

Ferroptosis is a unique form of cell death, induced by iron-mediated peroxidation of membrane lipids, which ultimately compromises membrane integrity [92]. It is regulated essentially by lipid metabolism, iron balance, redox status, and associated axes. The regulation of physiological and pathological states is critically dependent on ferroptosis, particularly in aging and oxidative stress. Moreover, it significantly impacts immune function by contributing to the death of leukocyte subsets, resulting in impaired immune responses [14, 92, 93].

Lipid peroxidation is critically involved in ferroptosis. The presence of excessive amounts of phospholipid hydroperoxides (PLOOH) in the cell membrane increases membrane tension. Subsequently, the influx of Ca^2^⁺ and Na⁺ is facilitated through the activation of ion channels, while K⁺ is simultaneously expelled. These ionic changes compromise intracellular equilibrium, inducing cell swelling and ultimately causing plasma membrane rupture [92]. Consequently, the production and modulation of PLOOH are closely linked to ferroptosis, with glutathione peroxidase 4 (GPX4) and system xc⁻ (a cystine/glutamate antiporter) serving as key regulatory components in this process [94–97]. GPX4 is the primary enzyme responsible for reducing PLOOH. It catalyzes the conversion of PLOOH into alcohols using electrons provided by glutathione (GSH). Inhibition of GPX4 by RSL3 results in a rapid accumulation of PLOOH in the presence of iron and ferrous ions, thereby inducing ferroptosis. System xc^–^ facilitates the transport of cysteine, a precursor for GSH synthesis, into the cell. Inhibition of system xc^–^ by Erastin reduces GSH synthesis, which indirectly promotes ferroptosis [14, 15, 92, 93, 98].

In the initial immune response during sepsis, the enhancement of macrophage bactericidal activity has been associated with ferroptosis inducers [99]. However, in Mycobacterium tuberculosis infection, macrophage death displays characteristics of ferroptosis. Further studies in mouse models have demonstrated that macrophage death is closely associated with downregulated GPX4 expression and ferroptosis induction [100]. Irisin can suppress inflammatory responses by protecting mitochondria and inhibiting ferroptosis. In the serum of sepsis patients, the level of irisin decreases and is negatively correlated with disease severity [101]. Evidence from these findings indicates that ferroptosis may function in two distinct ways within the immune system during sepsis. While it can reduce the numbers of immune cells, impairing immune function, it may also enhance their activity, improving pathogen clearance. Beyond its effects on immune cells, ferroptosis significantly contributes to tissue and organ damage in sepsis. Ferroptosis activation has been observed in sepsis mouse models and LPS-induced H9c2 myofibroblasts, and drug-induced blocking of ferroptosis reduces cardiac damage, suppresses inflammation, and extends the lives of LPS-treated mice [102]. Furthermore, ferroptosis has been implicated in atrial fibrillation by increasing atrial fragility, a process that can be mitigated by ferroportin (FPN) knockdown or ferroptosis inhibitors [103]. Thus, sepsis-related cardiac dysfunction and circulatory failure are critically influenced by ferroptosis occurring in cardiomyocytes [104].

Key regulators of ferroptosis include GPX4 and system xc^–^, which influence cellular outcomes as well as the immune network and organ functions by modulating intracellular iron and lipid metabolism [98, 105]. Within the immune system, ferroptosis has a dual impact: it can enhance the microbicidal activity of immune cells, thus boosting defense against infection, while excessive ferroptosis may impair immune system function, with decreases in both the immune cell numbers and efficacy [106, 107]. In sepsis-related organ damage, ferroptosis can contribute to a decline in cardiac function and arrhythmias, as well as the progression of complications such as SAE, SA-AKI, and sepsis-associated gastrointestinal injury. Consequently, precisely balancing the dual effects of ferroptosis on the pathophysiology of sepsis and further achieving precise spatiotemporal control of ferroptosis can enhance sepsis prevention and treatment, thereby significantly improving the prognoses and quality of life of sepsis patients.

### Sepsis-associated autophagy

Autophagy is a key controller of cellular homeostasis, primarily promoting cell survival under stress by facilitating the lysosomal degradation of harmful intracellular components. During nutrient scarcity, autophagy degrades organelles, proteins, lipids, and nucleic acids to generate energy for the cell [108]. Additionally, numerous genetic studies have clearly demonstrated that mutations in autophagy-related genes can lead to various human diseases, with autophagy and its associated cellular functions being widely implicated in the pathophysiological processes and mechanisms of these conditions [109].

The formation of autophagosomes represents a critical stage in the autophagy process. Based on specificity, autophagy is classified into two distinct types, namely, selective and non-selective autophagy [110]. Non-selective autophagy primarily occurs during cellular nutrient deprivation. In this context, isolation membranes or phagophores with a cup-shaped membrane structure are formed at the autophagosome formation site [111]. The ULK complex is activated at the endoplasmic reticulum and recruits ATG9 vesicles, which supply lipids to form the isolation membrane. The membrane then expands continuously, encapsulating cytoplasmic material into autophagosomes, which later integrate with lysosomes to generate autolysosomes for digestion [112, 113]. Selective autophagy, by contrast, targets specific substrates. In this process, receptor proteins with ubiquitin-binding domains and LC3-interacting region (LIR) domains act as bridges, recruiting to the site to link selective autophagy substrates that are ubiquitinated by E3 ubiquitin ligases with lipidated LC3/GABARAP proteins. The isolation membrane then expands and engulfs the substrates to form autophagosomes, which eventually fuse with autolysosomes [114, 115].

Studies have indicated that enhancing autophagy can significantly improve lung, liver, kidney, and cardiac injuries associated with sepsis [116–119]. In sepsis-associated lung tissue damage, the expression of autophagy-related proteins begins to decrease within 4 h of onset and remains low for up to 24 h. The experimental induction of autophagy can effectively decrease apoptosis and the production of pro-inflammatory cytokines, thus protecting lung tissue [120]. Similarly, enhanced autophagy was observed in liver tissues. Electron microscopy revealed that the number of autophagosomes in the livers of sepsis patients was markedly higher than that in the non—sepsis control group [121]. Blocking autophagy results in proximal tubular cell death during sepsis. CLP mice in the autophagy-inhibited group showed markedly higher mortality rates relative to the controls [122–124], indicating the involvement of autophagy in mitigating SA-AKI [88]. Regarding cardiac function, impaired autophagy can lead to contractile dysfunction and apoptosis in cardiomyocytes. Electron microscopy reveals a marked increase in autophagosomes, but a scarcity of autolysosomes in the later stages of sepsis. This blockage in autophagic flux suggests that the autophagy process in cardiomyocytes cannot proceed normally, resulting in the accumulation of damaged organelles and proteins, which ultimately disrupts normal cardiomyocyte function. Administration of rapamycin, an autophagy inducer, can restore autophagic flux integrity, promote the fusion of autophagosomes with lysosomes, improve cardiomyocyte function, restore left ventricular ejection fraction, and protect cardiomyocytes from apoptosis and necrosis [119, 125].

Cellular homeostasis is maintained through the crucial mechanism of autophagy. It involves the formation of vesicles at specific intracellular sites using lipids to selectively or non-selectively engulf cytoplasmic materials, inducing autophagosome formation and subsequent lysosomal fusion. This process facilitates the clearance of abnormal intracellular contents and helps maintain cellular nutritional status [126]. During the progression of sepsis, autophagy activation can reduce cellular death in tissues, preserve normal cellular functions, and reduce the production of pro-inflammatory factors.. As a result, it mitigates sepsis-associated organ damage and blocks the progression of multiple organ dysfunction syndrome (MODS) [127]. It is necessary to investigate controllable autophagy processes which will help improve patient survival and health. Table 1 provides a comprehensive synopsis of the influence of the five cell death types on target organs in sepsis.

**Table 1:** Mechanisms and effects of cell death in sepsis

Cell death pathway	Affected organs	Mechanism	Impact on sepsis	References
Apoptosis	Heart	Excessive apoptosis leads to a reduction in cardiomyocyte numbers, impairing both systolic and diastolic functions of the heart, which ultimately contributes to cardiac insufficiency. Moreover, the release of cellular contents during apoptosis further activates the inflammatory response, exacerbating myocardial damage	The release of inflammatory mediators and the enhancement of oxidative stress due to apoptosis intensify the pathological development of sepsis	[218, 219]
	Lung	The apoptosis of pulmonary microvascular endothelial cells disrupts the integrity of the pulmonary microvasculature, increasing vascular permeability. This process also compromises the alveolar epithelial cell barrier, allowing the infiltration of inflammatory cells and fluid into the alveolar cavity. Consequently, these alterations contribute to the development of pulmonary edema, non-cardiogenic pulmonary edema, and ARDS	Apoptosis disrupts the structures and functions of organs, further activates the immune system, and negatively affects the prognosis of sepsis	[220, 221]
	Kidney	In the kidneys, levels of pro-inflammatory cytokines and apoptosis-related proteins, such as BAX and cleaved caspase-3, are markedly increased, while those of anti-apoptotic proteins, including Bcl-2, are suppressed. The interplay between inflammation and apoptosis creates a vicious cycle that further exacerbates organ damage	Apoptosis not only worsens organ dysfunction but also drives the progression of chronic kidney disease while simultaneously activating a systemic inflammatory response	[222, 223]
	Liver	Apoptosis is associated with sepsis-induced liver damage by activating the TLR4/MyD88/NF-κB axis. This activation triggers a cascade of inflammatory responses, characterized by the release of pro-inflammatory cytokines such as TNF-α, IL-1β, and IL-6. These cytokines contribute to microvascular dysfunction and exacerbate organ damage, emphasizing the significant involvement of apoptosis in the pathogenesis of sepsis-associated liver injury	In sepsis, excessive apoptosis can influence the persistence of the inflammatory response, worsen organ damage, and induce immune cell dysfunction, thereby negatively impacting the prognosis	[224, 225]
Pyroptosis	Heart	Pyroptosis exacerbates local and systemic inflammatory responses by activating the NLRP3 inflammasome, impairing mitochondrial function and causing cell lysis. This process releases a large number of inflammatory factors, such as IL-1β and IL-18, which contribute to cardiomyocyte dysfunction and tissue damage	Pyroptosis accelerates the early deterioration of sepsis, promotes the development of MODS, and is closely linked to poor prognosis	[226–228]
	Lung	Caspase-3-mediated cleavage of GSDMD activates GSDMD-induced pores, promoting the release of inflammatory factors and inducing pyroptosis. This process directly damages lung tissue, resulting in pathological changes such as protein exudation and thickening of the alveolar septum. Consequently, these changes lead to the destruction of alveolar architecture, inflammatory cell infiltration, and pulmonary edema, thereby worsening ALI and ARDS	Pyroptosis releases inflammatory mediators and cellular contents that act as “danger signals.” These signals further activate the immune system, amplifying the inflammatory response and contributing to the development of MODS	[41, 229, 230]
	Kidney	THBS1 (thrombospongoprotein 1) promotes pyroptosis by activating the TGF-β/Smad3/NLRP3/caspase-1 axis, leading to renal tubular epithelial cell injury and death, disrupting renal tissue structure and function, as evidenced by increased serum creatinine and urea nitrogen levels, along with decreased urine output	Pyroptosis plays a significant role in promoting inflammation and injury in sepsis-induced AKI. Simultaneously, the interaction between pyroptosis and oxidative stress exacerbates cell and tissue damage	[231, 232]
	Liver	During sepsis, ROS levels in the liver are significantly elevated. PAMPs, such as LPS, can activate the NLRP3 inflammasome, thereby inducing pyroptosis. This process promotes the release of inflammatory mediators, exacerbating the inflammatory response in the liver and ultimately leading to hepatocyte injury and liver dysfunction	Pyroptosis-induced cell rupture and the subsequent release of inflammatory factors can trigger the activation of additional immune cells, thereby exacerbating the inflammatory response. This process may lead to SIRS, which further intensifies the severity of sepsis	[233, 234]
	Nervous system	Microglial pyroptosis releases substantial quantities of pro-inflammatory cytokines, such as IL-1β and IL-18, which exacerbate neuroinflammation. Neuroinflammation is a key pathological mechanism underlying SAE. Additionally, pyroptosis can induce neuronal death, further amplifying brain tissue damage. This process ultimately contributes to cognitive dysfunction and long-term neurological sequelae	Pyroptosis can compromise the integrity of the BBB, allowing additional inflammatory mediators and neurotoxins to infiltrate the central nervous system. This process further exacerbates nerve damage and inflammation, ultimately worsening the severity of sepsis	[235, 236]
	Bowel	The translocation of intestinal microorganisms and toxins into the bloodstream is facilitated by the compromised intestinal barrier, which results from the pyroptosis of intestinal epithelial cells and contributes to SIRS development. Moreover, pyroptosis can alter the intestinal microenvironment, leading to dysbiosis—an imbalance in the gut microbiota. This dysbiosis further weakens intestinal barrier function, increases intestinal permeability, and promotes the production of inflammatory factors, thereby establishing a vicious cycle. Concurrently, the intestinal mucosal immune system is overactivated in sepsis, resulting in the excessive release of inflammatory factors and exacerbating intestinal inflammation	A compromised intestinal barrier and the subsequent leakage of inflammatory mediators can initiate a systemic inflammatory response, leading to injury in essential organs like the lungs, liver, and kidneys. For instance, gut bacteria and toxins entering the bloodstream may induce inflammation in the pulmonary system, potentially progressing to ARDS	[237]
Necroptosis	Heart	In sepsis-induced myocardial injury, the activation of RIPK1, RIPK3, and MLKL constitutes a pivotal event in necroptosis. Upon activation, these molecules trigger cell membrane rupture, leading to the release of intracellular contents and exacerbating myocardial damage. The cardiomyocyte death and inflammation elicited by necroptosis can significantly worsen myocardial dysfunction, characterized by reduced myocardial contractility and cardiac output. This, in turn, further exacerbates circulatory failure in patients with sepsis	Necroptosis influences sepsis-induced myocardial injury and may potentially affect the progression of sepsis through the systemic inflammatory response. For example, DAMPs released during necroptosis can activate the immune system, exacerbating systemic inflammation	[218, 238]
	Lung	Necroptosis activates the RIP1/RIP3/mLKL axis, exacerbating inflammation and neutrophil infiltration. This process disrupts cell membrane integrity. Specifically, the release of intracellular contents destroys tight junction proteins between cells, such as ZO-1 and Occludin. This disruption increases the permeability of the alveolar-capillary barrier, resulting in pulmonary edema. Furthermore, necroptosis can further damage alveolar epithelial cells and vascular endothelial cells, ultimately impairing lung function	Necroptosis-induced cell membrane rupture and leakage of intracellular contents can disrupt tissues' normal structure and function. This disruption interferes with tissue repair and regeneration processes, prolonging recovery time for sepsis patients and increasing mortality rates	[239–241]
	Kidney	Necroptosis is a critical pathway in the pathogenesis of SA-AKI. The activation of necroptosis induces the death of renal tubular epithelial cells, leading to structural disruption and functional impairment of renal tissue. This compromises renal filtration and metabolic functions, thereby exacerbating the clinical condition of sepsis patients	DAMPs released during necroptosis can activate the immune system, triggering a systemic inflammatory response. This inflammatory cascade may exacerbate damage to other vital organs, including the lungs, liver, and heart	[88, 242]
	Liver	In septic liver injury, necroptosis activation significantly impairs the structural integrity and functional capacity of hepatocytes. Pathological changes, such as disordered hepatocyte arrangement, nuclear lysis, necrosis, and inflammatory cell infiltration, manifest as a result	During necroptosis, the release of intracellular contents, such as LDH, and the excessive production of inflammatory cytokines, including TNF-α and IL-1β, significantly amplify both local and systemic inflammatory responses	[243, 244]
Ferroptosis	Heart	Ferroptosis plays a critical role in sepsis-induced myocardial injury, characterized by elevated intracellular iron levels, depletion of GSH, and downregulation of GPX4 expression in cardiomyocytes. Ferroptosis induces cardiomyocyte damage and death, compromising the structural integrity and contractile function of the heart. This disruption in cardiac function ultimately leads to systemic circulatory dysfunction	Cardiac dysfunction resulting from ferroptosis can further compromise the blood supply and functional capacity of other organs, exacerbating the clinical condition of sepsis patients and contributing to an increased mortality rate	[245, 246]
	Lung	Under the influence of NETs, alveolar epithelial cells undergo ferroptosis, characterized by lipid peroxidation and elevated iron content. This process disrupts the structural and functional integrity of the alveoli, leading to pathological changes such as pulmonary edema, pulmonary hemorrhage, and hyaline membrane formation. These alterations significantly worsen the severity of ALI induced by sepsis	The activation of inflammatory cascades during ferroptosis is driven by the release of iron ions and lipid peroxidation products, which subsequently promote the secretion of pro-inflammatory cytokines such as TNF-α, IL-1α, IL-8, and TGF-β. This process significantly exacerbates SIRS	[246, 247]
	Kidney	In SA-AKI, ferroptosis is characterized by increased iron levels, accumulation of lipid peroxidation products (e.g., MDA), and reduced GPX4 expression in renal tubular epithelial cells. These changes impair cellular antioxidant defenses, leading to tubular necrosis, cast formation, and elevated renal function markers (creatinine and BUN)	Renal injury induces a systemic inflammatory response through the release of inflammatory factors, creating a vicious cycle of “renal injury-aggravated inflammation.” Additionally, abnormal lipid metabolism resulting from ferroptosis is associated with energy depletion and immunosuppression in sepsis patients, further reducing their chances of survival	[246, 248, 249]
	Liver	The expression of G protein-coupled receptor 116 (GPR116) is significantly upregulated in sepsis-induced liver injury. GPR116 inhibits the systemic Xc^–^/GSH/GPX4 axis, promoting ferroptosis and contributing to sepsis-associated acute liver injury	Liver injury can exacerbate the inflammatory response and disrupt hepatic detoxification, metabolism, and immune function via the gut-liver axis. This disruption delays the recovery of liver function, negatively impacting overall therapeutic efficacy in sepsis and further intensifying the severity of the condition	[246, 250, 251]
	Nervous system	Nerve cell damage and death occur due to ferroptosis, a regulated process of cell death associated with iron-dependent lipid peroxidation. This process disrupts normal nervous system function. Recent studies have shown a close correlation between sepsis-induced ferroptosis and neuronal damage, particularly in the hippocampus. Such damage can lead to cognitive dysfunction, decreased learning and memory abilities, disturbances in consciousness, and other related symptoms	Damage to the nervous system impairs neurological function and may also negatively affect the function of other organs through the neuro-endocrine-immune network. For example, neural injury can lead to autonomic dysfunction, affecting other vital organ systems such as the cardiovascular and respiratory systems. This, in turn, may exacerbate sepsis-induced MODS	[246, 252]
Autophagy	Heart	Autophagy plays a complex dual role in sepsis-induced cardiac injury. On one hand, autophagy protects cardiomyocytes by clearing damaged organelles and protein aggregates and modulating oxidative stress and inflammation. However, excessive autophagy activation may cause cardiomyocytes to undergo autophagic cell death, exacerbating cardiac injury	Autophagy-induced damage to cardiomyocytes impairs both systolic and diastolic cardiac function, directly compromising the heart's ability to pump effectively. During sepsis, the heart is required to increase its output to meet heightened systemic metabolic demands. However, the decline in cardiac function caused by autophagy-mediated cardiomyocyte injury makes the heart incapable of effectively meeting these demands, exacerbating the clinical condition of sepsis patients	[218, 253]
	Lung	Platelet-derived exosomes can induce the autophagy process in neutrophils, promoting NET release. The accumulation of NETs in the lungs causes damage to alveolar epithelial and endothelial cells. Additionally, circulating mtDNA activates the STING pathway, leading to autophagy dysfunction. This dysfunction contributes to severe lung tissue damage, including alveolar capillary congestion, pulmonary interstitial edema, and extensive inflammatory cell infiltration	Excessive activation of autophagy can lead to a reduction in cell numbers. The release of extracellular matrix components during autophagy may alter the lung tissue microenvironment, affecting cell adhesion and migration. These changes can impede the repair process of lung tissue and subsequently influence patient prognosis	[207, 254, 255]
	Kidney	The role of autophagy in SI-AKI is akin to a “double-edged sword.” It can protect cells from injury or lead to cell death when over-activated. In renal tubular epithelial cells (RTECs), autophagy levels initially increase transiently following septic stimulation but then sharply decline	Sepsis-induced kidney injury often leads to renal dysfunction. This dysfunction results in the accumulation of metabolic waste, electrolyte disturbances, and fluid imbalances, further worsening of the systemic inflammatory response can contribute to the development of multiple organ dysfunction	[256–258]
	Liver	Disruption of the autophagy process in liver tissue, including obstruction of autophagy progression and impairment of autophagy flux, leads to significant liver injury. This injury is manifested as severe hepatocyte necrosis, pronounced inflammatory cell infiltration, and hepatic steatosis	Hepatic injury leads to the release of numerous inflammatory mediators, exacerbating the systemic inflammatory response. Furthermore, dysfunction of hepatic detoxification and metabolic regulation amplifies the systemic inflammatory response and increases the risk of multiple organ dysfunction	[207, 259]

## The role of cell death in the pathophysiology of sepsis

### Crosstalk between cell death and inflammatory responses

The inflammatory response serves as a defining feature of sepsis, with its magnitude and spread shaping disease progression and outcome. The various cell death pathways actively engage with inflammatory mechanisms during sepsis [128, 129]. As well as causing pro-inflammatory cytokine production, their activation also influences upstream and downstream molecular and cellular components of the inflammatory cascade, playing a pivotal role in its regulation [130]. Given the significance of these interactions, this review systematically evaluates how different cell death processes are linked to inflammation in sepsis. The clarification of these processes can lead to the identification of potential therapeutic strategies that can modulate the inflammatory response to an optimal level—preventing tissue and organ damage while effectively clearing invading pathogens. This approach would be beneficial for patients with sepsis.

**Fig. 2:**
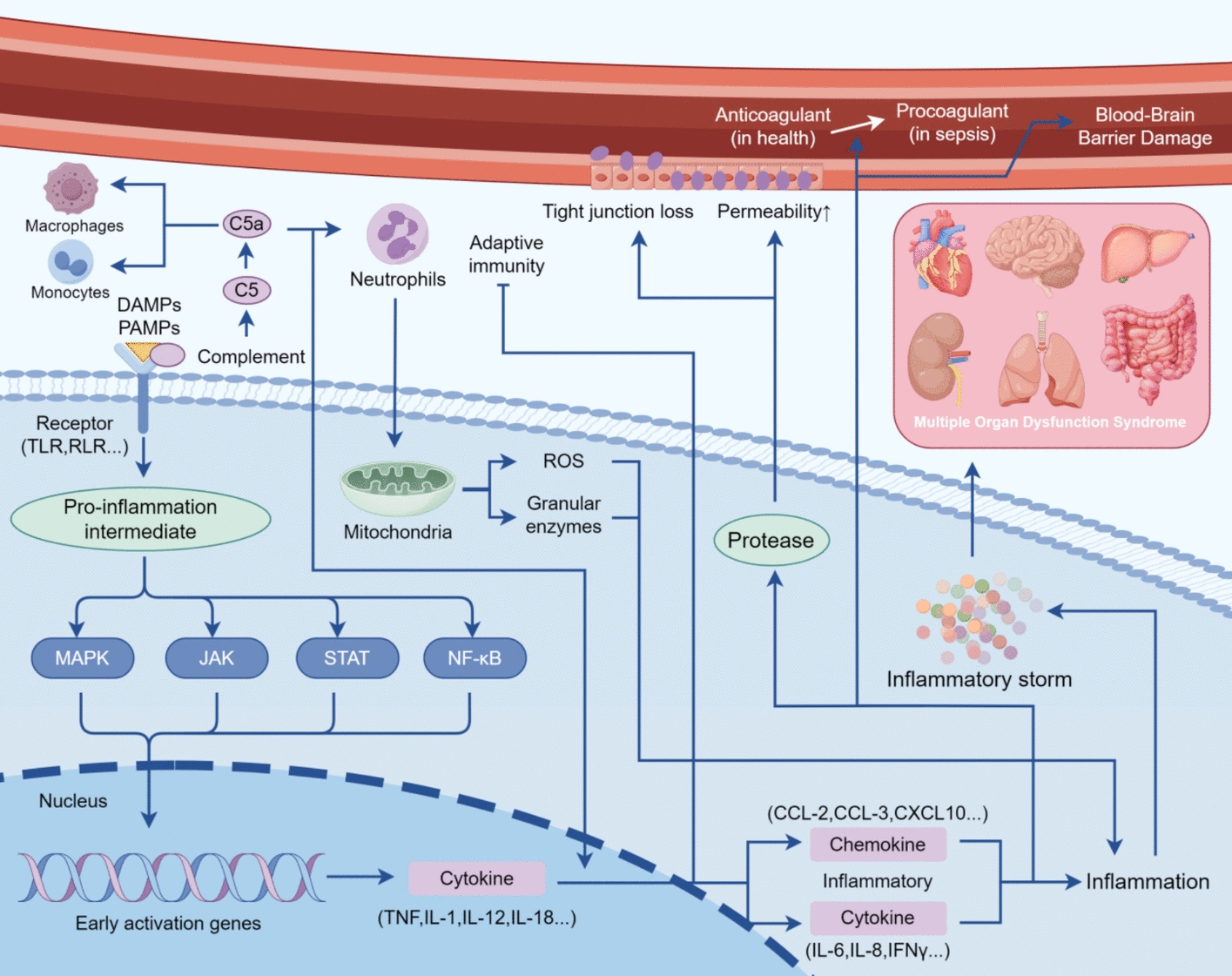
Inflammatory cascade involved in sepsis. The initiation of the inflammatory cascade in sepsis is typically triggered by the recognition of DAMPs and PAMPs by cell surface receptors, with contributions from the complement system. These receptors are expressed on various cell types, including immune, epithelial, and endothelial cells, which collectively serve to monitor and recognize their surrounding microenvironment. Upon activation, these receptors induce the downstream activation of pro-inflammatory intermediates, such as MAPK, JAK, STAT, and NF-κB. Following the activation of the inflammatory signal, the expression of early activation genes is induced, leading to the production of inflammation-related cytokines. These cytokines not only initiate a cascade of downstream inflammatory cytokines and chemokines but also facilitate the polarization and suppression of the adaptive immune system. Inflammatory molecules and cells interact and crosstalk with one another, culminating in a robust chain reaction that ultimately results in an inflammatory storm. This systemic inflammatory cascade can affect the entire body, contributing to the development of MODS. In addition to their role in mediating inflammation, these cytokines and chemokines significantly impact the vascular endothelium, the coagulation system and blood–brain barrier. It is also crucial to note that the complement system plays a key role in inflammatory pathways. Upon activation, the complement system generates complement peptides, with C5a being the most potent. C5a activates macrophages and monocytes and stimulates neutrophils to undergo oxidative bursts, further amplifying the inflammatory response. (By Figdraw)

On pathogen invasion, PRRs of the immune system recognize PAMPs, resulting in the upregulation of inflammatory factors and stimulation of their downstream pathways [131, 132]. If pathogens are successfully cleared, anti-inflammatory mechanisms regulate and counteract the inflammatory response. However, if microbial virulence is excessive or host immunity is insufficient, PAMPs persist and continuously stimulate PRRs, resulting in excessive inflammation. The anti-inflammatory response may also become dysregulated, ultimately causing tissue and organ damage, leading to sepsis [133]. In addition to exogenous PAMPs, cell damage and sustained excessive inflammation can produce DAMPs, such as histones, dsDNA, and heat shock proteins (HSPs). These molecules can also activate PRRs, eliciting similar responses and further promoting sepsis development [134]. Activation of upstream pathways mediates the expression of various inflammatory mediators. Specifically, recognition of PAMPs and the activation of DAMPs results in the recruitment of pro-inflammatory intermediates, which activate the phosphorylation of mitogen-activated protein kinases (MAPKs), Janus kinases (JAKs), or signal transducers and activators of transcription (STAT), as well as the nuclear translocation of nuclear factor-κB (NF-κB), triggering early response gene expression [2]. The inflammatory mechanism during sepsis is depicted in Fig. 2. Cell death pathways are engaged at each stage of the sepsis inflammatory response, resulting in either the amplification or suppression of inflammation. Advancing knowledge of these pathways will improve the ability to design strategies for modulating inflammation in sepsis.

Inflammatory responses are significantly influenced by pyroptosis, which is one of the key cell death pathways [135, 136]. The excessive immune response in a cytokine storm results in systemic hyperinflammation, a life-threatening condition involving the overexpression of inflammatory mediators in the circulation [137]. Research findings suggest that cytokine storms may worsen due to the heightened levels of pyroptosis in innate immune cells [138–140]. Specifically, DAMPs, IL-1β, and IL-18 released during pyroptosis can further upregulate inflammatory mediators, such as IL-6, IL-8, and TNF [138, 141, 142]. The IL-1 family is crucial in inflammation and the immune response. Pyroptosis-related molecules, including caspases-1, –4, and –11, are responsible for activating IL-1β and IL-18, which are then transferred with GSDMD-associated pores, along with IL-1α and IL-33 [143, 144]. Additionally, IL-1 can synergize with TNF to cause tissue damage, further exacerbating the severity of sepsis [145]. Pyroptosis can also result in the release of high-mobility group box 1 (HMGB1), which functions as a DAMP and binds to other pro-inflammatory molecules, such as DNA, histones, IL-1α, and IL-1β. This interaction enhances intestinal cell permeability, disrupts gut barrier function in mice, and contributes to the progression of intestinal inflammation [145–148]. In addition to these molecules, pyroptosis also releases galectin-1, ATP, SQSTM1, and other molecules that function as DAMPs, promoting macrophage polarization, among other effects [138, 145, 149–151].

In addition to pyroptosis, other cell death pathways also contribute significantly to upstream inflammatory signaling. In an ALI mouse model resulting from LPS stimulation, IL-6 and TNF-α levels in the bronchoalveolar lavage fluid (BALF) were substantially increased. However, exposure to Fer-1 to inhibit ferroptosis markedly reduced the IL-6 and TNF-α contents of the BALF, suggesting a close association between ferroptosis and pro-inflammatory factors [152]. Severe inhibition of neutrophil apoptosis is strongly linked to the onset of sepsis. Under harmful stimuli, the number and survival time of neutrophils in the bloodstream increase rapidly. Activated neutrophils then migrate and release large amounts of cytokines. Anti-apoptotic environments containing neutrophils may contribute markedly to systemic inflammation and organ injuries, although persistent survival of neutrophils may be an adaptation to infection [25, 26, 153, 154]. Autophagy can alleviate overly intense inflammatory responses by degrading pro-inflammatory cytokines, including IFN-γ, TNF-α, and interleukins, including IL-1α, IL-1β, IL-33, and IL-36. Additionally, impaired autophagy promotes NLRP3 inflammasome overactivation, which subsequently induces excessive inflammation [110, 155–157]. Necroptosis disrupts cell membrane integrity during sepsis progression, enabling release of inflammatory mediators, including HSPs and HMGB1. This mechanism enhances the secretion of significant quantities of cytokines and chemokines, including IL-6, CXC chemokine ligand 1 (CXCL1), CXCL2, CC chemokine ligand (CCL8), and CCL2, which in turn stimulate inflammatory and immune responses [158, 159]. Notably, necroptosis can act synergistically with GSDMD-induced pyroptosis, promoting inflammation and inducing tissue injury [9]. The impact of cell death on inflammation during sepsis is summarized in Table 2**.**

**Table 2:** The impact of various cell death modalities on inflammation during sepsis

Cell death	Effect	Mechanism	Subject of study	References
Apoptosis	Pro-inflammation	TUNEL staining and flow cytometry demonstrated a significant increase in the apoptosis rate of mouse cardiomyocytes and H9C2 cells induced by LPS. This increase in apoptosis was closely linked to the upregulation of inflammatory factors, including IL-6, IL-1β, and TNF-α	H9C2 cardiomyocytes, LPS-induced sepsis mice models	[260]
		Apoptosis in endothelial cells can impair vascular barrier function, leading to vascular leakage and tissue damage, which further exacerbates the inflammatory response	Human umbilical vein endothelial cells (HUVECs)	[261]
		DAMPs released by apoptotic cells activate macrophages, such as THP-1 cells, prompting them to secrete higher levels of inflammatory factors, including IL-1β, IL-6, and TNF-α, exacerbating the systemic inflammatory response	Macrophages (THP-1 cells), alveolar epithelial cells (BEAS-2B cells), human lung microvascular endothelial cells (HLMVECs), CLP-induced sepsis mice models	[262]
		LPS-stimulated RTECs release proinflammatory cytokines during apoptosis, intensifying inflammation and attracting additional immune cells, such as neutrophils and macrophages, to infiltrate the kidney and further amplify the inflammatory response	HK-2 cells, TCMK-1 cells, CLP-induced sepsis mice models	[263]
		EVs released by apoptotic cells may contain pro-inflammatory microRNAs, such as miR-155, which can be taken up by macrophages. This uptake activates the NF-κB axis, promoting the secretion of pro-inflammatory cytokines, such as TNF-α and IL-6, further exacerbating the inflammatory response	Macrophages, Endothelial cells, Mesenchymal stem cells (MSCs)	[264]
	Anti-inflammation	Neutrophils can be engulfed by macrophages via apoptosis, limiting the persistence and spread of the inflammatory response. Simultaneously, macrophages secrete anti-inflammatory factors, such as TGF-β1, which further inhibits the inflammatory response	Patients with sepsis or septic shock, PD-L1 knockout neutrophils	[26]
		Extracellular vesicles released by apoptotic macrophages, termed apoptotic extracellular vesicles (apoEVs), significantly reduce the sepsis-associated inflammatory response. This reduction is accompanied by improved tissue damage and enhanced survival rates in infected mice	S. aureus-induced sepsis mice models	[265]
		MicroRNA-146a (miR-146a) present in EVs released by apoptotic cells can inhibit the NF-κB axis in macrophages, thereby reducing the secretion of pro-inflammatory cytokines	Macrophages, Endothelial cells, MSCs	[264]
Pyroptosis	Pro-inflammation	During pyroptosis, HMGB1 is released in a GSDMD-dependent manner, specifically through cell rupture rather than the GSDMD pore. This release allows HMGB1 to function as a DAMP, activating receptors on immune cells and enhancing the inflammatory response	LPS-induced macrophage and hepatocyte, patients with sepsis and septic shock	[266]
		Pyroptosis exacerbates the inflammatory response in sepsis through activation of NLRP3 inflammasomes and caspase-1-dependent pathways, leading to the release of proinflammatory cytokines such as IL-1β and TNF-α	Patients with sepsis and septic shock, CLP-induced sepsis mice models	[267]
		Pyroptosis in RTECs exacerbates renal inflammation through activation of the NLRP3 inflammasome, leading to the release of inflammatory factors and infiltration of inflammatory cells	HK-2 cells, ACSS2 knockout mice	[232]
		Upon LPS stimulation of endothelial cells, the NLRP3 inflammasome is activated, subsequently leading to the activation of caspase-1. The activation of caspase-1 results in the maturation and release of inflammatory factors such as IL-1β and IL-18, thereby exacerbating the inflammatory response	LPS induced human umbilical vein endothelial cells (HUVEC) and human aortic endothelial cells (TeloHAEC)	[268]
		Pyroptosis facilitates the propagation of GSDMD pores via extracellular vesicles (EVs), triggering bystander cell death and thereby exacerbating the inflammatory response. This mode of transmission resembles a domino effect, leading to widespread cell death and the diffusion of inflammation	Mouse bone marrow-derived Macrophages (BMDMs), Gsdmd −/− mice, Nlrp3 −/− mice, Casp11 −/− mice	[269]
	Anti-inflammation	Pyroptosis in large peritoneal macrophages (LPMs) releases inflammatory mediators, such as IL-1β, which attract additional immune cells, including monocytes, neutrophils, and B1 cells, leading to the formation of multicellular aggregates known as resMf-aggregates. These aggregates accumulate on the surface of mesothelial cells in the peritoneal cavity, immobilized by fibrin networks, forming dynamic immune cell scaffolds that facilitate bacterial clearance and localized control of inflammation	LPMs in mice	[270]
Necroptosis	Pro-inflammation	Necroptosis induces cell membrane rupture via the RIPK1-RIPK3-MLKL pathway, leading to the release of DAMPs such as HMGB1, ATP, and histones into the extracellular space. These DAMPs activate PRRs on immune cells, triggering downstream cascades and promoting the release of inflammatory factors, including IL-1β and IL-18, further exacerbating the inflammatory response	Patients with sepsis and septic shock, CLP-induced sepsis mice models	[266]
		In the necroptosis pathway, RIPK3 inhibits the autophagic degradation of STING, maintaining the activation of the STING axis. Activation of the STING pathway leads to the release of inflammatory factors, such as IFNβ and TNF-α, which in turn further activate necroptosis, creating a positive feedback loop that exacerbates the inflammatory response	HT-2 cells, RAW264.7 cells, HEK293T cells, THP-1 cells, BMDMs, CLP-induced sepsis mice models, patients with sepsis and septic shock	[271]
		The formation of the NLRP3 inflammasome is facilitated by necroptosis, which subsequently induces GSDMD-mediated membrane pore formation. This leads to the release of inflammatory mediators, including IL-1β, thereby enhancing immune cell infiltration and intensifying the inflammatory response	LPS-Induced sepsis mice models	[238]
Ferroptosis	Pro-inflammation	The inflammatory factors IL-1β, TNF-α, and IL-6 are produced as a result of ferroptosis. Through autocrine and paracrine signaling, these factors activate inflammatory pathways, including NF-κB and JNK, which further promote the secretion of inflammatory mediators and contribute to inflammatory cascade development. Moreover, these inflammatory factors enhance the absorption and utilization of intracellular iron ions, further exacerbating ferroptosis	H9c2 cells, AC16 cells, LPS-induced sepsis mice models	
		During ferroptosis, intracellular iron ion levels rise, leading to enhanced lipid peroxidation and the production of large amounts of ROS. These ROS can directly damage cells, activate inflammatory axes, and activate M1 macrophages. The release of numerous pro-inflammatory cytokines, including IL-1β and TNF-α, is triggered by this activation, thereby intensifying the inflammatory response	LPS-Induced sepsis mice models	[272]
		PTGS2, a key enzyme in ferroptosis, exhibits significantly elevated expression levels. By catalyzing the conversion of arachidonic acid to prostaglandins, PTGS2 further promotes inflammation	LPS-induced neonatal rat cardiomyocytes (NRCMs), LPS-induced sepsis mice models	[273]
		In sepsis-induced myocardial injury, ferroptosis significantly elevates intracellular ROS levels. These ROS induce lipid peroxidation, leading to the production of harmful substances such as 4-hydroxynonenal (4-HNE) and MDA. These substances compromise cell membrane integrity and activate various inflammatory axes, including the Nrf2 pathway	LPS-induced sepsis mice models	[274]
	Anti-inflammation	The inhibition of DC maturation and activation by ferroptosis is evident from the downregulation of co-stimulatory molecules, reduced cytokine secretion, and impaired T cell proliferation. This suggests a regulatory role of ferroptosis in suppressing the inflammatory response in sepsis	CLP-induced sepsis mice models	[275]
		The transition of macrophages from the pro-inflammatory M1 phenotype to the anti-inflammatory M2 phenotype during sepsis is regulated by ferroptosis, contributing to the suppression of the inflammatory response	Low doses of polymicrobial infection of Hmox1 knockout mice	[276]
Autophagy	Pro-inflammation	Excessive activation of autophagy exacerbates the inflammatory response and renal injury via the JNK/p38-ATF2 axis. The increased secretion of inflammatory factors, including TNF-α, IL-1β, and IL-6, is also a consequence of this overactivation, further amplifying the inflammatory response	LPS-induced sepsis mice models, LPS-induced HK-2 cells	[277]
	Anti-inflammation	Autophagy exerts anti-inflammatory effects primarily through Nrf2-mediated macrophage polarization and the NF-κB/PPARγ axis. Activation of Nrf2 enhances autophagy and modulates macrophage polarization towards an anti-inflammatory phenotype, thereby mitigating lung injury and the inflammatory response associated with sepsis	Patients with sepsis and septic shock, CLP-induced sepsis mice models	[278]
		Autophagy mitigates ROS production by eliminating damaged mitochondria and degrades components of the NLRP3 inflammasome, inhibiting the inflammatory response. Additionally, autophagy regulates the secretion of inflammatory cytokines, reducing the release of pro-inflammatory cytokines, such as TNF-α and IL-6, thereby dampening the inflammatory response	LPS-Induced HK-2 cells, LPS-induced sepsis mice models	[279]
		By modulating intracellular metabolism and energy status, autophagy can influence the intensity of the inflammatory response. For instance, autophagy can attenuate excessive inflammatory responses by regulating the AMPK and mTOR axes	CLP-induced sepsis mice models	[157]
		Autophagy promotes macrophage polarization from the pro-inflammatory M1 phenotype to the anti-inflammatory M2 phenotype. This process enhances macrophage phagocytosis of apoptotic neutrophils, reducing the accumulation of inflammatory cells and the release of inflammatory factors. Additionally, autophagy can modulate the miRNA composition in EVs secreted by human MSCs, enhancing neutrophil phagocytic activity and macrophage efferocytosis, facilitating inflammation resolution	hMSCs, CLP-induced sepsis mice models	[280]

In sepsis, the relationship between cell death and inflammatory responses is bidirectional. Cell death can initiate inflammatory processes by releasing cytokines and chemokines that drive inflammation, and MAPK and NF-κB, among other downstream pathways, undergo activation [160]. Conversely, various inflammatory factors and pathways can induce cell death [161]. Since multiple pathways and mechanisms are associated with cell death, and inflammatory responses are modulated by different cell death pathways, the regulatory network is intricate and highly interconnected. For instance, the activation of one pathway may concurrently suppress others, further complicating research in this field [162]. To optimize the modulation of inflammatory responses and enhance therapeutic efficacy, it is crucial to consider the various cell death pathways as an integrated system and identify key molecules that regulate the role of cell death in inflammation. This approach may lead to the discovery of one or more emerging therapeutic strategies that can control various cell death pathways and appropriately modulate the intensity of inflammatory responses in sepsis. Such strategies would not only improve the management of sepsis and patient outcomes but also enhance the survival and health of patients with sepsis.

### Impact of cell death on immunosuppression

During the onset and progression of sepsis, immune suppression is a key factor contributing to adverse outcomes, characterized primarily by immune cell exhaustion and reprogramming of antigen-presenting cells [132, 133]. Different cell death pathways are associated with immunosuppression. These pathways not only induce immune suppression by lowering the number of immune cells but also indirectly impair immune function by altering the epigenetic modifications of these cells [163]. Although anti-inflammatory mechanisms continuously function in sepsis patients to regulate inflammation, promote tissue repair, and restore homeostasis, dysregulated inflammatory responses may ultimately result in persistent immunosuppression, increasing the likelihood of secondary infection [164]. Through antigen presentation by DCs, B lymphocytes, and T lymphocytes, the adaptive immune system is stimulated in conjunction with the innate immune system, promoting the generation of pathogen-specific antibodies and memory cells. However, as sepsis progresses, the function of antigen-presenting cells declines, reducing antigen presentation capacity and further exacerbating immune suppression [133]. Therefore, a more thorough understanding of the factors driving sepsis-induced immune suppression is critical for improving prognosis and reducing mortality.

In sepsis patients, apoptosis levels of CD4^+^ and CD8^+^ T cells, B cells, and DCs are markedly elevated, and the functions of CD4^+^ T helper 1 (Th1), Th2, and Th17 cells are reduced [165]. Furthermore, sepsis patients show decreased HLA-DR on blood monocytes and impaired ability of monocytes and macrophages to secrete pro-inflammatory cytokines, a condition termed “immune paralysis” or “LPS tolerance.” In sepsis, HLA-DR expression on DCs is also reduced, while apoptosis levels of conventional and plasmacytoid DCs are increased. Experimental studies have indicated that reducing DC apoptosis can improve survival rates in sepsis [166, 167]. Tregs exhibit higher resistance to sepsis-induced apoptosis due to intracellular anti-apoptotic proteins, and their survival levels remain relatively higher in sepsis patients. Tregs exert their effects by suppressing the function and proliferation of effector T cells, monocytes, and neutrophils or by interacting with monocytes via the Fas/FasL pathway and directly engaging with DCs. They downregulate co-stimulatory molecule expression and induce lymphocyte anergy, thereby worsening immune suppression [163, 165, 168, 169]. Blocking Treg activity has been shown to improve immune function and increase microbial killing capacity in sepsis, highlighting the suppressive role of Tregs [170]. In addition to apoptosis, other cell death pathways contribute to reduced immune cell numbers. For example, the pathogens *Staphylococcus aureus* and *Streptococcus pneumoniae* can release pore-forming toxins that induce necroptosis in macrophages, worsening sepsis outcomes [171–173]. During sepsis, normal autophagy programs can provide protection by detoxifying harmful substances, maintaining mitochondrial function, and regulating cytokine release [174]. However, abnormal autophagy states may be detrimental; for instance, blocking autophagy in CD4^+^ T cells increases IL-10 secretion, which inhibits differentiation of IFN-γ-producing Th1 cells and natural killer (NK) cell activation in sepsis [175]. Reduced monocyte levels contribute significantly to immune suppression, and ferroptosis driven by solute carrier family 39 member 8 (SLC39A8) is implicated in this process. Inhibition of SLC39A8 reduces LPS-induced lipid peroxidation [176]. In myeloid cells, the conditional knockdown of Gpx4 enhances caspase-11 activation and GSDMD cleavage in a lipid peroxidation-dependent manner, resulting in macrophage pyroptosis [177]. Overall, both innate and adaptive immune cells are activated by sepsis stimuli, resulting in cell death through various pathways. This either directly inhibits immune function or releases signals that suppress other immune cells, ultimately paralyzing the immune system. Addressing pathogen immune evasion, preventing continuous stimulation of immune cells by pathogens, and mitigating immune suppression induced by cell death represent critical therapeutic strategies for preventing sepsis from becoming severe.

**Fig. 3:**
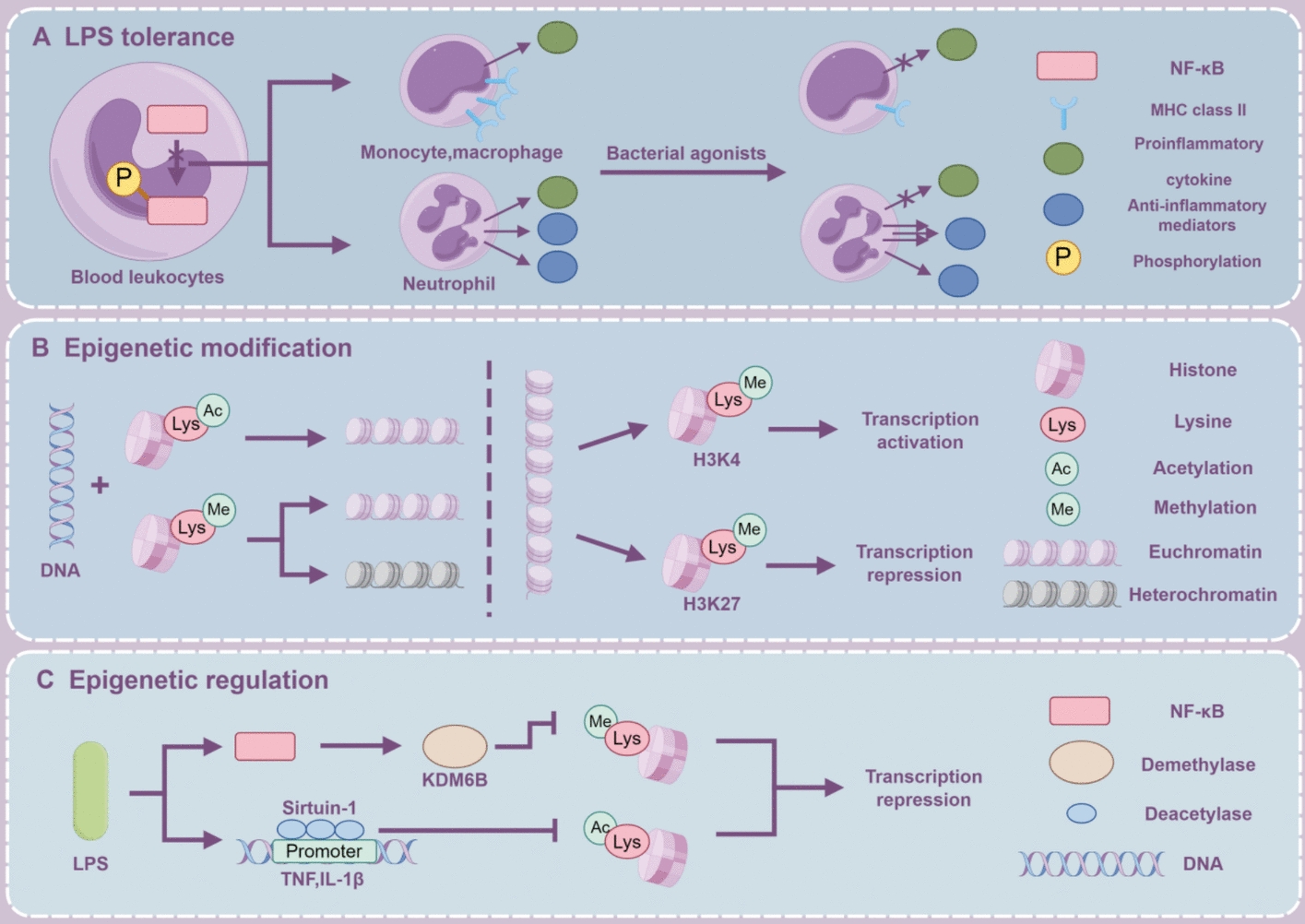
Reprogramming and epigenetic mechanisms of immune cells. **A** Impaired phosphorylation of NF-κB may contribute to the development of LPS tolerance in blood leukocytes. Upon stimulation by bacterial agonists, there is a reduction in the expression of MHC class II molecules on the surface of monocytes and macrophages, along with a decreased production of pro-inflammatory cytokines. Similarly, neutrophils show a reduced expression of pro-inflammatory cytokines, while the expression of anti-inflammatory mediators either increases or remains unchanged. **B** The epigenetic mechanism underlying LPS tolerance involves the modification of histone proteins. Generally, the acetylation of histone lysines promotes the formation of heterochromatin, whereas the methylation of lysines can lead to either euchromatin or heterochromatin formation, depending on the specific lysines modified. Among the various histone modifications, the methylation of histone 3 lysine-4 (H3K4) and histone 3 lysine-27 (H3K27) is closely associated with transcriptional activation and repression, respectively. **C** The stimulation of LPS activates NF-κB in immune cells, inducing the expression of the demethylase KDM6B and recruiting the deacetylase sirtuin-1 to the promoters of TNF and IL-1β, thereby suppressing the transcriptional expression of pro-inflammatory mediators. (By Figdraw)

Sepsis-induced immune suppression prominently involves reprogramming antigen-presenting cells, influencing the function of myeloid cells through epigenetic regulation of gene expression. During this process, chromatin gene loci are placed into active or silent states, often through histone modifications such as acetylation, methylation, ubiquitination, and phosphorylation [178]. In sepsis, epigenetic regulatory mechanisms can be disrupted, resulting in an immunosuppressive phenotype in immune cells. Research has shown that histone modifications underlie LPS-induced tolerance in monocytes [179]. For example, increased levels of repressive histone modifications are observed in the promoters regions of IL-1β and TNF following LPS stimulation of macrophages [180, 181]. Similar observations have been made in LPS-stimulated macrophages or monocytes derived from sepsis patients [181]. Molecular mechanisms include LPS-induced upregulation of the histone lysine demethylase KDM6B (JMJD3) in macrophages through NF-κB signaling [182]. Moreover, the TNF and IL-1β promoter regions accumulate histone deacetylase sirtuin 1 (SIRT1) as a result of LPS stimulation [183]. Current evidence suggests that the impact of cell death on immune cell epigenetics is more likely mediated through shared molecular or pathway overlaps (e.g., NF-κB, IL-1β) rather than direct epigenetic regulation by cell death. To date, no studies have elucidated how cell death directly influences epigenetic regulation in immune cells. This area presents a promising avenue for future research. This approach may broaden the applicability of epigenetic therapies in treating sepsis and other diseases. Figure 3 illustrates the mechanisms of reprogramming and epigenetic regulation in immune cells.

Overall, the functioning of the immune system is markedly affected by cell death during sepsis [133]. This process transitions from initial immune activation to subsequent passive suppression, a shift often marking disease deterioration and the onset of sepsis-induced chronic immunosuppressive condition (PICS) [184]. The specific mechanisms by which different cell death modes contribute to immune suppression can be summarized as follows: immune cells involved in the immune response, upon undergoing various cell death pathways, not only directly reduce immune function through their own death but also alter cell phenotypes, induce further cell death, and suppress immune activity by releasing a range of signaling molecules. This cascade ultimately leads to immune suppression [185]. Consequently, developing unique and effective treatment methods for sepsis must focus on addressing the reduction in immune system function, further preventing and treating adverse outcomes in sepsis patients, reduce severity ratios among patients, and substantially alleviate the overall medical burden on both patients and society.

## Targeting cell death pathways for therapeutic benefit

### Therapeutic strategies for distinct modes of cell death

The pathogenesis, progression, and prognosis of sepsis are critically influenced by cell death pathways [186]. Targeting these pathways offers promising opportunities for innovative sepsis treatment strategies that could reverse poor prognoses and benefit patient outcomes [184]. In addition, extensive interactions and signaling crosstalk occur between different cell death pathways, significantly influencing sepsis pathophysiology [187]. Thus, therapeutic approaches should not target only a single cell death pathway. Instead, it is necessary to implement a comprehensive approach that integrates the interdependence of multiple cell death pathways [188]. The crosstalk between these diverse pathways is summarized in Table 3. By taking this integrated approach, it becomes possible to more effectively modulate cell death processes, optimizing therapeutic efficacy while reducing potential adverse effects. This comprehensive treatment strategy not only has the potential to improve the outcomes of patients with sepsis but also offers new perspectives for addressing other complex diseases.

**Table 3:** Crosstalk between different cell death pathways

Cell death I	Cell death II	Molecule	Mechanism	In vivo or in vitro	References
Apoptosis	Pyroptosis	Caspase-8	Caspase-8 is a key protease in the initiation of apoptosis. Additionally, it facilitates pyroptosis through the cleavage of GSDMD, particularly during TNF-induced inflammation	In vivo and in vitro experiments	[47, 281]
		GSDMD	During pyroptosis, GSDMD is cleaved by caspase-1 or caspase-11, leading to the formation of cell membrane channels. These channels facilitate cell lysis and the subsequent release of inflammatory factors. In apoptosis, GSDMD can also be cleaved by caspase-3, particularly under specific infection conditions	In vitro experiments	[47]
		Caspase-1	Caspase-1 is a crucial executor of pyroptosis. It directly cleaves caspase-7, a key molecule in apoptosis, thereby activating its function. This interaction establishes a link between pyroptosis and apoptosis	In vivo and in vitro experiments	[281]
		ASC	ASC functions as an inflammasome component in pyroptosis, linking caspase-1 to other inflammatory signaling molecules. In apoptosis, ASC interacts with the DED domain of caspase-8 via its PYD domain	In vivo and in vitro experiments	[281]
		Pannexin-1	During pyroptosis, Pannexin-1 forms a pore upon activation, increasing cell membrane permeability. This process leads to the release of cellular contents and the subsequent activation of the inflammatory response. Conversely, in apoptosis, Pannexin-1 activation may accelerate apoptosis by enhancing cell membrane permeability	In vivo and in vitro experiments	[282]
Apoptosis	Necroptosis	ZBP1	ZBP1 is an essential sensor for influenza virus-induced NLRP3 inflammasome activation. Additionally, ZBP1 plays a role in regulating the apoptotic pathway through its interaction with caspase-8	In vivo and in vitro experiments	[283]
		RIPK3	RIPK3 is a key kinase involved in necroptosis regulation. Upon interacting with ZBP1, RIPK3 phosphorylates MLKL, which subsequently triggers necroptosis. Additionally, RIPK3 interacts with caspase-8, thereby contributing to apoptosis regulation	In vivo and in vitro experiments	[283]
		Caspase-8	Caspase-8, a critical enzyme in apoptosis, can be activated by ZBP1 to initiate apoptosis. Besides its role in apoptosis, caspase-8 also regulates necroptosis by inhibiting RIPK3-mediated necroptotic signaling	In vivo and in vitro experiments	[283, 284]
		RIPK1	RIPK1 interacts with FADD to recruit and activate caspase-8, thereby initiating apoptotic signaling. Concurrently, RIPK1 interacts with RIPK3 to activate MLKL, leading to membrane rupture and necroptosis	In vivo and in vitro experiments	[284]
Pyroptosis	Necroptosis	RIPK3	In necroptosis, RIPK3 forms a complex with RIPK1 to activate MLKL, inducing membrane disruption. In the pyroptosis pathway, RIPK3 promotes NLRP3 inflammasome activation by engaging caspase-8, ultimately leading to pyroptosis	In vitro experiments	[47]
		MLKL	Following phosphorylation by RIPK3, MLKL oligomerizes and translocates to the cell membrane in necroptosis, forming pores that increase membrane permeability and ultimately cause cell death. In pyroptosis, MLKL can also activate the NLRP3 inflammasome, promoting the maturation and release of inflammatory factors such as IL-1β	In vivo and in vitro experiments	[285]
		GSDMD	In pyroptosis, GSDMD forms pores in the cell membrane, leading to cell swelling and lysis. This process facilitates the release of inflammatory factors, thereby initiating an inflammatory response. In necroptosis, pore formation by GSDMD causes potassium efflux, inhibiting cGAS-STING pathway activation and reducing IFN production, which regulates the intensity of inflammation	In vitro experiments	[286]
Pyroptosis	Ferroptosis	GSDME	During pyroptosis, caspase-3-mediated cleavage of GSDME results in pore formation in the cell membrane, leading to cellular content release and immune system activation. In ferroptosis, GSDME expression levels influence cellular sensitivity, with high GSDME expression increasing ferroptosis susceptibility, while low expression may confer resistance	In vivo and in vitro experiments	[287]
		ROS	ROS promote lipid peroxidation, inducing ferroptosis and causing cell membrane damage. Additionally, ROS activate the NLRP3 inflammasome, leading to caspase-1 activation and pyroptosis induction	In vivo and in vitro experiments	[285]
Necroptosis	Ferroptosis	Caspase-8	Caspase-8 suppresses necroptosis by inhibiting RIPK1 and RIPK3 activity. Furthermore, caspase-8 indirectly modulates ferroptosis by maintaining intracellular NADPH levels	In vivo and in vitro experiments	[288]
Necroptosis	Autophagy	STING	In autophagy, STING interacts with p62 (SQSTM1), directing it to autophagosomes for degradation. This process prevents excessive activation of the cGAS-STING axis, functioning as a negative feedback mechanism. In necroptosis, STING activation promotes IFN and TNF production, leading to RIPK1 and RIPK3 activation. These kinases phosphorylate and activate MLKL, thereby inducing cell death	In vitro experiments	[286]
		RIPK1/RIPK3	In necroptosis, RIPK1 and RIPK3 function as upstream kinases that initiate necroptosis through MLKL phosphorylation. Their activation is closely associated with oxidative stress signals, particularly ROS generated by mitochondria. These ROS modulate autophagy intensity, influencing the cellular response to necroptotic stimuli	In vivo and in vitro experiments	[289]
		CAMKII	RIPK3 activates calcium/calmodulin-dependent protein kinase II (CAMKII) in necroptosis, regulating the mitochondrial permeability transition pore (mPTP). This process leads to mitochondrial membrane potential loss and subsequent cell death In autophagy, CAMKII activation is associated with mitochondrial dysfunction, influencing autophagy regulation	In vivo and in vitro experiments	[290]
		p53	The tumor suppressor protein p53 regulates autophagy-related gene expression in autophagy. Similarly, in necroptosis, p53 modulates the expression of necroptosis-related genes, affecting cell death pathways	In vivo and in vitro experiments	[290]
Ferroptosis	Apoptosis	GSDMD	During apoptosis, GSDMD is cleaved by caspase-3, releasing its N-terminal pore-forming domain, which inserts into the membrane to form pores, leading to cell lysis and intracellular content release. In ferroptosis, lipid peroxidation increases membrane permeability, and GSDMD pore formation may exacerbate this effect, promoting cell death	In vivo and in vitro experiments	[14]
		GPX4	GPX4 prevents ferroptosis by scavenging intracellular lipid peroxides. GPX4 degradation leads to lipid peroxide accumulation, inducing ferroptosis. Additionally, GPX4 degradation promotes apoptosis by upregulating early growth response 1 (EGR1), which activates apoptosis-related proteins such as Bax while inhibiting anti-apoptotic proteins like Bcl-2 and Bcl-xL, thereby inducing apoptosis	In vivo and in vitro experiments	[284, 291]
Ferroptosis	Autophagy	NCOA4	Nuclear receptor coactivator 4 (NCOA4) functions as a selective autophagy receptor, binding to ferritin and directing it to autophagosomes for degradation. Ferritin degradation releases iron ions, increasing intracellular iron levels, a key trigger for ferroptosis. By promoting ferritin degradation, NCOA4 indirectly facilitates ferroptosis	In vitro experiments	[285]
		RAB7A	RAB7A, a small GTPase involved in vesicle trafficking, regulates lipid droplet metabolism, indirectly inhibiting lipid peroxidation and suppressing ferroptosis	In vitro experiments	[285]
		p62	p62 (SQSTM1), an autophagy receptor protein, binds ubiquitinated proteins and directs them to autophagosomes for degradation. It also mediates ferritin and GPX4 degradation, regulating intracellular lipid peroxidation and ferroptosis	In vitro experiments	[285]
		AMPK	During energy deficiency, AMPK activation promotes autophagy to remove damaged mitochondria and excess ferritin, maintaining cellular energy balance. Additionally, AMPK regulates lipid metabolism, influencing lipid peroxidation levels and ferroptosis	In vitro experiments	[285]
Autophagy	Apoptosis	PINK1	The interaction between PINK1 and Beclin1 initiates autophagosome formation and exerts anti-apoptotic effects. A decrease in PINK1 levels facilitates the transition from autophagy to apoptosis	In vitro experiments	[292]
		Beclin1	Beclin1, a crucial regulator of autophagosome formation, interacts with proteins such as Vps34 to facilitate autophagosome maturation. Additionally, Beclin1 binds Bcl-2 family proteins, including Bcl-2 and Bcl-xL, via its BH3 domain. This interaction counteracts Bcl-2 anti-apoptotic functions, promoting apoptosis	In vivo and in vitro experiments	[293]
		JNK	JNK promotes autophagy by activating Beclin1, whereas in apoptosis, JNK activates pro-apoptotic proteins such as c-Jun and BIM	In vitro experiments	[294]
		ATG5	ATG5, a key autophagosome formation protein, can be cleaved and translocated to mitochondria, where it interacts with Bcl-xL to trigger cytochrome c release and caspase activation, facilitating apoptosis	In vitro experiments	[294]
		p53	p53 promotes autophagy by regulating autophagy-related genes, such as DRAM1. In apoptosis, p53 activates pro-apoptotic proteins, including Bax and PUMA	In vivo and in vitro experiments	[294]
		HMGB1	HMGB1 binds BECN1, relieving BCL2-mediated autophagy inhibition and promoting autophagy. The oxidative status of HMGB1 influences apoptosis regulation, with oxidized HMGB1 triggering caspase-dependent apoptosis through the mitochondrial pathway	In vivo and in vitro experiments	[295]
Autophagy	Pyroptosis	TRAF6	TRAF6 activation enhances NLRP3 inflammasome assembly and activation, promoting pyroptosis, while also exerting an inhibitory effect on autophagy	In vitro experiments	[296]
		miR-146a-5p	miR-146a-5p inhibits TRAF6 expression by targeting its mRNA, reducing NLRP3 inflammasome activation and suppressing pyroptosis. miR-146a-5p upregulation also promotes Beclin1 expression, enhancing autophagy	In vivo and in vitro experiments	[296]
		ROS	Moderate ROS levels inhibit pyroptosis by activating autophagy to clear damaged organelles, while excessive ROS activate inflammasomes, promoting pyroptosis	In vivo and in vitro experiments	[297]
		CTSB	Moderate ROS levels inhibit pyroptosis by activating autophagy to clear damaged organelles, while excessive ROS activate inflammasomes, promoting pyroptosis	In vivo and in vitro experiments	[286]

The modulation of apoptosis is primarily aimed at addressing immune suppression caused by a direct reduction in immune cell numbers and organ damage resulting from tissue cell apoptosis. Targets for intervention within the apoptotic pathway include apoptosis-related death receptors, pro- and anti-apoptotic factors, and caspase proteins. For instance, melatonin has demonstrated protective effects against sepsis by activation of the PI3K/Akt axis [189, 190]. This mechanism involves reducing serum and cardiac TNF-α levels, lowering oxidative stress, and suppressing both extrinsic and intrinsic apoptotic pathways. Reducing the levels of pro-apoptotic proteins while enhancing those of their anti-apoptotic counterparts accompany these effects, which collectively reduce inflammatory responses and alleviate organ damage [191]. Additionally, caspase inhibitors, such as zVAD.fmk and L-826,791 (M-791), effectively prevent lymphocyte apoptosis, thereby enhancing immune function and improving survival in sepsis patients [192].

The caspase family, inflammasomes, and gasdermin family are key components of the pyroptosis pathway, and targeting these molecules can effectively regulate pyroptosis, thereby modulating inflammatory levels in the body [62, 193]. For instance, the broad-spectrum caspase inhibitor VX-166 has been shown to alleviate pyroptosis by decreasing caspase-1 activity, lowering IL-1β and IL-18 with potent anti-inflammatory effects [194]. Similarly, through direct interaction with the Cys191 site of GSDMD, necrosulfonamide (NSA) inhibits the oligomerization of GSDMD-N dimers, thereby preventing GSDMD-induced pore formation and ultimately reducing the mortality of LPS-treated septic mice. This action suppresses pyroptosis and reduces pro-inflammatory cytokines in monocytes/macrophages of sepsis patients [195].

Key molecules, including RIPK1, RIPK3, and MLKL, along with the formation of complex IIb (the necrosome), are critical in the necroptosis pathway, and therapeutic approaches targeting these molecules have become a focus of research. In sepsis mouse models, Nec-1 inhibits necroptosis by reducing RIPK1 activity, leading to decreased serum levels of IL-6, IL-1β, and IL-18, as well as downregulation of neutrophil chemoattractants and macrophage inflammatory protein 2. These effects alleviate lung injury and improve survival rates in mice [196]. NSA covalently binds to cysteine residues of MLKL, inhibiting its activity and blocking necrosome assembly and maturation, which protects cells from necroptosis [197, 198]. Importantly, necroptosis mediated by RIPK1 and RIPK3 significantly contributes to the increased mortality observed during sepsis. This effect can be mitigated by caspase-8, which cleaves RIPK1 and RIPK3 to inhibit necroptosis. Consequently, the use of caspase-8 activators may be an approach for targeting necroptosis [84]. In contrast, pan-caspase inhibitors or selective caspase-8 inhibitors may induce necroptosis, potentially worsening sepsis outcomes [199].

The regulation of ferroptosis is primarily dependent on the balance between lipid peroxidation, associated proteins, and iron levels [200]. Uridine, a potential agent for preventing sepsis-induced ALI, exerts its effects through the activation of the antioxidant system. It enhances the expression of nuclear factor E2-related factor 2 (Nrf2) and its target genes (e.g., ACSL4) in lung tissue and macrophages, elevates GSH levels, and reduces the synthesis of lipid peroxidation byproducts, including malondialdehyde (MDA), therefore preventing ferroptosis [201, 202]. Furthermore, following uridine treatment, levels of pro-inflammatory cytokines, including TNF-α, IL-1β, and IL-6, are significantly reduced, indicating its anti-inflammatory properties [202]. Dexmedetomidine (Dex), a commonly used anesthetic agent, protects against vascular leakage during sepsis by modulating metabolic reprogramming to inhibit ferroptosis. Dex not only elevates GPX4 expression but also reduces iron concentrations and pro-inflammatory cytokine expression, thereby offering cardioprotective effects by mitigating ferroptosis [203].

During autophagy, autophagosomes and lysosomes are essential regulatory molecules. Nitric oxide (NO), a crucial immunomodulatory molecule, can induce ROS-mediated autophagy by eliminating mitochondrial ROS (mtROS) and mtDNA. This process prevents the excessive activation of the NLRP3 inflammasome and preserves mitochondrial functional stability. Thus, the use of NO inducers may promote autophagy and enhance cell survival during sepsis [204, 205]. Sinomenine facilitates the conversion of LC3-I to LC3-II in liver and lung cells, initiating autophagosome formation and thereby enhancing autophagy, which improves survival rates in septic mice [206]. Erbin, an inflammatory response regulator, promotes lysosome biogenesis by directly targeting transcription factor EB (TFEB), enhancing autophagy, reducing inflammatory responses, and alleviating organ damage [207]. Overall, as autophagy primarily offers protective effects in sepsis, therapeutic strategies targeting autophagy typically involve promoting the expression or assembly of autophagy-related molecules. A comprehensive summary of drugs influencing cell death pathways is provided in Table 4.

**Table 4:** Therapeutic drugs targeting cell death pathways in sepsis

Drug	Cell death involved	Targeted molecule or cell	Effect on target	Mechanism	References
NAD (H)-loaded nanoparticles	Apoptosis, pyroptosis	NAD (H)	Promotion	NAD (H) nanoparticles directly replenish cellular NAD(H) levels and inhibit apoptosis and pyroptosis by suppressing the NF-κB axis and NLRP3 inflammasome activation. Additionally, these nanoparticles protect vascular endothelial function and enhance cellular energy supply	[261]
Mesenchymal stem cell-derived apoptotic vesicles (apoVs)	Apoptosis	Fas/FasL pathway	Promotion	ApoVs induce neutrophil apoptosis by activating the Fas receptor via FasL. By shifting neutrophil death from NETosis to apoptosis, apoVs reduce neutrophil infiltration into distal organs such as the lung, spleen, and liver, mitigating inflammation and organ damage, thereby improving survival in septic mice	[298]
TSPO-PROTAC	Apoptosis	The mitochondrial translocator protein (TSPO)	Inhibition	TSPO-PROTAC selectively degrades the TSPO protein by recruiting intracellular E3 ubiquitin ligase, reducing phosphorylation and oligomerization of VDAC. This process decreases cytochrome c and mtDNA release caused by increased mitochondrial membrane permeability, leading to reduced caspase-3 activation and apoptosis. Additionally, TSPO-PROTAC reduces inflammatory factor expression, including TNF-α, IL-1β, and IL-6, thereby alleviating inflammation	[299]
Sialic acid-modified liposomal doxorubicin (DOX-SAL)	Apoptosis	Neutrophil	Promotion	DOX-SAL is taken up by neutrophils through a specific endocytic pathway, subsequently degraded in lysosomes, releasing DOX. The released DOX inserts into DNA, induces DNA damage, and activates the apoptotic pathway. By decreasing inflammatory neutrophil numbers, DOX-SAL significantly reduces neutrophil infiltration at inflammatory sites and lowers inflammatory factor release, such as TNF-α and IL-1β	[300]
β-Nicotinamide Mononucleotide (NMN)	Apoptosis	NAD +/SIRT1 axis	Promotion	NMN replenishes NAD^+^ levels and activates the NAD^+^-dependent deacetylase SIRT1, which inhibits p38 MAPK and NF-κB p65 phosphorylation through deacetylation. This process reduces inflammation, decreases sepsis-induced neuronal apoptosis, and improves SAE	[301]
Puerarin	Apoptosis	PGAM5-VDAC1 axis	Inhibition	Puerarin targets the PGAM5-VDAC1 axis to reduce mitochondrial membrane permeability, preventing mitochondrial dysfunction and apoptosis. Additionally, puerarin decreases apoptosis-related protein expression, including caspase-3 and caspase-9, thereby inhibiting apoptosis	[302]
IL-7,IL-15	Apoptosis	Anti-apoptotic protein	Promotion	IL-7 and IL-15 prevent immune cell apoptosis by upregulating anti-apoptotic proteins such as Bcl-2. These interleukins increase IFN-γ levels, enhancing immune cell pathogen clearance while reducing Treg numbers, thereby decreasing immune suppression	[303]
NAD + and BAPTA-AM co-loaded, acid-responsive ultrasmall hollow mesopore polydopamine nanoparticles (HMPDA@BA/NAD)	Apoptosis, pyrotosis	Ca^2+^, NLRP3 inflammasome	Inhibition	(1) NAD^+^ inhibits NLRP3 inflammasome activation, reducing IL-1β and IL-18 production, effectively suppressing pyroptosis. (2) BAPTA-AM prevents Ca^2+^ overload-induced mitochondrial membrane potential collapse and cytochrome c release by chelating excess Ca^2+^. Concurrently, NAD^+^ restores mitochondrial function and reduces apoptosis-related protein activation, including caspase-9 and caspase-3, thereby inhibiting mitochondrial apoptosis	[139]
Fibroblastic reticular cell-derived exosomes (FRC-Exos)	Pyroptosis	NLRP3 inflammasome	Inhibition	FRC-Exos are specifically taken up by renal tubular epithelial cells. By promoting mitophagy, these exosomes reduce NLRP3 inflammasome activation, thereby decreasing pro-inflammatory cytokine release, such as IL-1β and IL-18. This suppression mitigates inflammation and pyroptosis, ultimately alleviating SI-AKI	[304]
Ticagrelor	Pyroptosis	ASC	Inhibition	Ticagrelor inhibits ASC oligomerization by blocking chloride efflux, preventing NLRP3 inflammasome activation. This inhibition reduces pyroptosis and decreases pro-inflammatory cytokine secretion, including IL-1β and TNF-α	[305]
4-hydroxynonenal (HNE)	Pyroptosis	NLRP3 inflammasome	Inhibition	HNE directly binds to NLRP3, inhibiting inflammasome activation, thereby suppressing pyroptosis and reducing IL-1β and IL-18 maturation and release	[306]
Z-VAD-FMK, Ac-FLTD-CMK, VX-765	Pyroptosis	Caspase-1,-4,-5,-11	Inhibition	Several drugs target caspases, inhibiting their activity and preventing GSDMD-induced pore formation, thereby suppressing pyroptosis	[41]
Disulfiram (DSF)	Pyroptosis	TLR4	Inhibition	By modifying MD-2, a key TLR4 cofactor, DSF blocks TLR4-mediated activation of NF-κB and IRF3 axes, inhibiting pyroptosis and reducing IL-1β, IL-6, and TNF-α production	[307]
NU6300	Pyroptosis	GSDMD	Inhibition	NU6300 inhibits GSDMD activation by covalently binding to cysteine 191 on GSDMD via its vinyl sulfone group, preventing GSDMD cleavage by inflammatory caspases. Additionally, NU6300 inhibits GSDMD palmitoylation, reducing its effective localization to the cell membrane and preventing functional pore formation, thereby inhibiting pyroptosis	[308]
Necrostatin-1	Necroptosis	RIPK1	Inhibition	Nec-1 blocks RIPK1 autophosphorylation, inhibiting necroptosis. In sepsis-induced cardiomyopathy, Nec-1 significantly reduces myocardial cell death and improves cardiac function	[218]
Serine incorporator 2 (Serinc2)	Necroptosis	Akt axis	Promotion	Serinc2 modulates GSK-3β phosphorylation by activating the Akt axis, thereby inhibiting RIPK3-MLKL complex formation and suppressing necroptosis	[309]
Linifanib	Necroptosis	RIPK1	Inhibition	Linifanib directly inhibits RIPK1 kinase activity and prevents its phosphorylation, thereby suppressing necrosis. Additionally, linifanib prevents RIPK1 binding to TNFR1, inhibiting necrosome formation and reducing inflammation	[310]
ZB-R-55	Necroptosis	RIPK1	Inhibition	ZB-R-55 functions as a dual-mode inhibitor, binding both the allosteric and ATP-binding pockets of RIPK1. By engaging the allosteric pocket, ZB-R-55 modulates RIPK1 conformation, reducing its activity, effectively blocking necroptosis, and diminishing inflammation, demonstrating significant therapeutic efficacy in sepsis models	[311]
Dexmedetomidine (Dex)	Necroptosis	MLKL, NLRP3 inflammasome	Inhibition	Dex inhibits MLKL phosphorylation and NLRP3 inflammasome formation, suppressing necroptosis and reducing inflammation. Additionally, Dex inhibits JNK phosphorylation, modulating inflammatory responses and fibrosis	[312]
GSK872	Necroptosis	RIPK3	Inhibition	GSK872 inhibits RIPK3 kinase activity and reduces MLKL phosphorylation, blocking downstream necroptosis signaling and decreasing cerebrovascular endothelial cell death	[313]
Oleanolic Acid (OA)	Ferroptosis	Acyl-CoA Synthetase Long-Chain Family Member 4 (ACSL4)	Inhibition	OA reduces ACSL4 expression, decreasing lipid peroxide production and alleviating ferroptosis-induced cellular damage	[314]
Ferrostatin-1 (Fer-1)	Ferroptosis	Sideroflexin 1 (SFXN1), NCOA4	Inhibition	Fer-1 inhibits NCOA4 activity, reducing ferritin degradation and decreasing intracellular iron ion release, thereby mitigating ferroptosis. Additionally, Fer-1 inhibits the mitochondrial membrane protein SFXN1, reducing mitochondrial iron overload and ROS production	[102]
Dexrazoxane (DXZ)	Ferroptosis	Fe^2+^, Prostaglandin endoperoxide synthase 2 (PTGS2)	Inhibition	DXZ alleviates ferroptosis by chelating intracellular iron ions, reducing their availability. Additionally, it inhibits PTGS2 expression, decreasing lipid peroxidation product formation	[102]
Irisin	Ferroptosis	GPX4	Inhibition	GPX4 upregulation inhibits ferroptosis, reducing sepsis-induced liver injury. GPX4 lowers lipid peroxidation and protects cells from ferroptosis-related damage	[101]
Artesunate	Ferroptosis	Nrf2/HO-1 pathway	Inhibition	Nrf2 upregulates antioxidant enzyme expression, reducing oxidative stress. Artesunate inhibits ferroptosis by activating the Nrf2/HO-1 pathway, thereby reducing sepsis-induced lung injury	[104]
Rapamycin	Ferroptosis	mTOR	Inhibition	mTOR inhibition decreases iron accumulation and lipid peroxidation, thereby inhibiting ferroptosis. Rapamycin regulates iron metabolism and the antioxidant system by inhibiting the mTOR axis, alleviating sepsis-induced brain injury and cognitive impairment	[104]
α-CD200R antibody	Autophagy	CD200R	Promotion	The α-CD200R antibody restores neutrophil autophagy by blocking CD200-CD200R interactions. Additionally, this antibody inhibits IGF-1-mediated Treg generation, alleviating immunosuppression	[315]
Rapamycin	Autophagy	mTOR	Inhibition	mTOR is a key regulator of cell growth and metabolism, and its inhibition relieves autophagy suppression, promoting autophagosome formation and enhancing autophagy. In SA-AKI, rapamycin protects the kidney by activating autophagy, reducing cell damage, and diminishing inflammation	[279]
Sirtuin 6	Autophagy	p53	Promotion	Sirtuin 6 deacetylates p53, enhancing its activity and promoting autophagosome formation and autophagy. This process facilitates the removal of damaged organelles and protein aggregates, reducing cell damage and inflammation, thereby alleviating SA-AKI	[279]
Fibroblast growth factor 21 (FGF21)	Autophagy	mTOR axis, hypoxia-inducible factor-1α (HIF-1α)	Inhibition	FGF21 inhibits inflammation and reduces sepsis-induced liver injury by suppressing the mTOR axis, restoring autophagic flux, and promoting p62-dependent autophagic degradation of HIF-1α	[316]
Carbon monoxide (CO)	Autophagy	Autophagy-related genes and proteins	Promotion	CO enhances cellular stress resistance by activating autophagy-related genes, such as Beclin1, and proteins, such as LC3B-II. This activation promotes autophagosome formation and the degradation of damaged proteins and organelles. CO-induced autophagy regulates inflammation by reducing neutrophil infiltration, lowering inflammatory cytokine release, and promoting macrophage-mediated phagocytosis of apoptotic neutrophils, facilitating inflammation resolution	[280]
Exosomes derived from bone MSCs (BMSCs-Exo)	Autophagy	AMPK/mTOR axis	Promotion	BMSCs-Exo activates the AMPK/mTOR axis, increases the LC3-II/LC3-I ratio, and reduces p62 protein expression, restoring autophagic flux and exerting a protective effect in sepsis-induced AKI	[317]

In the pathophysiology of sepsis, various modes of cell death exhibit distinct characteristics; however, their underlying mechanisms are all closely linked to excessive inflammation and immunosuppression. To effectively design multidimensional therapeutic approaches for sepsis, it is essential not to simply restrict the occurrence of different forms of cell death. Instead, based on a comprehensive analysis of multiple cell death pathways, a holistic therapeutic strategy should be developed, with the central focus being the use of a combination approach to modulate multiple cell death mechanisms simultaneously. The objective of this strategy is to reduce excessive inflammation without inducing immunosuppression, maintaining immune function while preventing an overly strong inflammatory response. Furthermore, therapeutic plans must be personalized based on the stage and severity of sepsis, as well as the specific needs of the patient. As therapeutic strategies targeting these cell death pathways continue to evolve and improve, the prognosis of patients with sepsis is anticipated to achieve substantial amelioration.

### Current clinical transformation challenges in sepsis therapy

Although many therapeutic approaches have been identified, the research findings on sepsis that can be truly translated into clinical applications remain limited. The number of successful translations of therapeutic research from in vivo or in vitro models into clinical use is far from sufficient. Among the diverse modes of cell death, including apoptosis and pyroptosis, the relationship between sepsis and apoptosis stands out as particularly noteworthy and deserving of focused investigation. The pore formation in the membrane mediated by pyroptosis and the release of inflammatory factors are key factors leading to excessive inflammation and immune dysfunction in sepsis. Therefore, we will focus on the therapeutic effects of pyroptosis-targeted strategies for sepsis and their prospects for clinical translation.

A novel alarmin/PRR-targeting system, whose core is the TLR4/MD2/RAGE-blocking peptide (TMR peptide) based on the HMGB1 interaction domain, has recently been developed. During sepsis, the interaction between alarmins (e.g., HMGB1, PTX3) and multiple PRRs (e.g., TLR4, MD2, RAGE) activates caspase-11-mediated pyroptosis. The intracellular substances (e.g., DAMPs) released by pyroptosis exacerbate the pathological process of sepsis and worsen the patient's prognosis. Moreover, pyroptosis can enhance the alarmin-PRR-mediated pro-inflammatory signaling pathway, creating a vicious cycle of inflammation amplification [208–211]. Notably, these alarmins play a key role in the late stage of sepsis, offering a crucial time window for clinical intervention [212]. To improve the pharmacokinetics of the TMR peptide, researchers coupled it with liposomes to create TMR-Lipo. Experiments showed that TMR-Lipo effectively suppresses TLR4 and RAGE-mediated inflammation in LPS-stimulated macrophages. Significantly, when combined with antibiotics, TMR-Lipo markedly enhances survival rates in CLP-induced sepsis mouse models [213]. However, the system's specificity and selectivity for the host immune system remain unclear, and the animal models used have limitations. Additionally, key information on its potential off-target effects, pharmacokinetics, in vivo safety, and clinical feasibility is still insufficient. Thus, extensive research is needed to evaluate its safety and efficacy before clinical application.

In addition to targeting the alarmin/PRR system, current therapeutic strategies also focus on molecules like IL and NLRP3, but these have yet to reach clinical application. Most previous models have concentrated on acute injuries to single systems, such as ALI and AKI. There are still many unresolved issues regarding the specificity, effectiveness, and safety of various treatments that need in-depth exploration. Future research will focus on several key areas. First, it will involve more precise and individualized selection of immune targets to enhance treatment accuracy. Second, it will address the challenges of translating preclinical research into clinical use. Third, it will comprehensively evaluate potential therapeutic candidates. Lastly, it will explore innovative strategies to bridge the gap between research and clinical practice, aiming to steadily advance the clinical application of research in this field.

## Conclusion

In the context of sepsis, a complex interaction exists between cell death signaling, inflammatory responses, and the immune system, emphasizing the need for innovative therapeutic approaches to address this intricate scenario. Recent years have seen substantial progress in knowledge of different cell death types, including apoptosis, pyroptosis, necroptosis, ferroptosis, and autophagy. These modes of cell death are closely linked to sepsis pathogenesis and progression and contribute to the development of sepsis-induced chronic immunosuppressive condition (PICS).

An examination of cell death mechanisms reveals that these processes are not only outcomes of cellular damage but also serve as initiating factors for various immune responses, significantly affecting the balance between inflammation and its resolution. During cell death, the release of DAMPs and PAMPs triggers a cascade of immune activation events. These events can, in turn, positively feedback to activate other cell death pathways, leading to the widespread release of pro-inflammatory molecules such as cytokines and complement. If uncontrolled, this process may result in cytokine storms, systemic hyperinflammation, and multiple organ dysfunction. However, extensive cell death may also lead to immune cell exhaustion or phenotypic changes, resulting in immunosuppression. This paradox between immunosuppression and excessive inflammation increases the likelihood of secondary infections. Therefore, in sepsis pathophysiology, cell death mechanisms not only trigger inflammatory responses but also drive immune dysfunction. A comprehensive understanding of these processes is essential for the development of targeted therapeutic strategies.

Research into cell death pathways provides a unique perspective for enhancing sepsis treatment. Therapies that modulate apoptosis, pyroptosis, necroptosis, ferroptosis, and autophagy can offer dual benefits by improving pathogen clearance while preserving immune function. Traditionally, these forms of cell death have been viewed as independent processes. However, increasing evidence has shown that, in the context of sepsis, these cell death pathways are linked with excessive inflammation and immunosuppression, often interacting in ways that are difficult to separate within the disease [177, 199, 214]. For instance, the activation of one inflammatory pathway typically involves multiple cell death pathways, and the suppression of a specific immune cell type may also engage several cell death pathways [47, 215–217]. Drug interventions targeting a single form of cell death may unintentionally impact other forms, reducing therapeutic efficacy or even causing adverse reactions. Therefore, by integrating the regulatory roles of various cell death pathways in excessive inflammation and immunosuppression during sepsis, and identifying key “convergence points” (such as caspase family, gasdermin family, NF-κB pathway etc.), the efficacy of sepsis treatment can be improved while reducing adverse reactions. This study proposes a comprehensive reassessment of the role of cell death in the pathophysiological mechanisms of sepsis by focusing on shared pathways and molecular targets involved in these processes, more effective sepsis therapies can be developed. However, it must be acknowledged that the development of sepsis therapies targeting cell death is still in its infancy. There are significant gaps in clinical data regarding the assessment of the strengths and weaknesses of animal models, as well as the efficacy and safety of drug use. Future research urgently requires investigators to delve deeper into these areas and refine therapeutic strategies in order to surmount the numerous challenges that lie between experimental research and clinical application.

In summary, a deeper understanding of the interplay between cell death pathways, inflammation, and immunity enhances knowledge of sepsis pathophysiology and provides a foundation for developing novel therapeutic strategies. Ongoing research in this area holds the potential to fundamentally transform the approach to sepsis treatment, bringing us closer to improving clinical outcomes for this life-threatening condition. By optimizing therapeutic strategies, the goal is to improve patient outcomes globally and reduce the significant burden sepsis places on healthcare systems worldwide.

## Data Availability

No datasets were generated or analysed during the current study.

## Abbreviations

AKI: Acute kidney injury
ALI: Acute lung injury
Apaf-1: Apoptotic protease activating factor 1
AMPK: AMP-activated protein kinase
AMs: Alveolar macrophages
ARDS: Acute respiratory distress syndrome
ASC: Apoptosis-associated speck-like protein
ATG13: Autophagy-related protein 13
BALF: Bronchoalveolar lavage fluid
BBB: Blood–brain barrier
CCL: CC chemokine ligand
CIRP: Cold-inducible RNA-binding protein
CLP: Cecal ligation and puncture
COX-2: Cyclooxygenase-2
CXCL: CXC chemokine ligand; DAMPs: damage-associated molecular patterns
DCs: Dendritic cells
DIC: Disseminated intravascular coagulation
Dex: Dexmedetomidine
dsDNA: Double-stranded DNA
FADD: Fas-associated protein with death domain
FPN: Ferroportin
GAPDH: Glyceraldehyde-3-phosphate dehydrogenase
GPX4: Glutathione peroxidase 4
GSDMC: Gasdermin C
GSDMD: Gasdermin D
GSDME: Gasdermin E
GSH: Glutathione
HMGB1: High-mobility group box 1
HSPs: Heat shock proteins
IFN: Interferon
IL-6: Interleukin-6
iNOS: Inducible nitric oxide synthase
IRF3: Interferon regulatory factor 3
JAKs: Janus kinases
JNK: c-Jun n-terminal kinase
LDH: Lactate dehydrogenase
LIR: LC3-interacting region
LKB1: Liver kinase B1
LPS: Lipopolysaccharide
MAPKs: Mitogen-activated protein kinases
MDA: Malondialdehyde
MDDCs: Monocyte-derived DCs
MLKL: Mixed lineage kinase domain-like
mtDNA: Mitochondrial DNA
mtROS: Mitochondrial ROS
MLCK: Myosin light chain kinase
MSCs: Mesenchymal stem cells
mTORC1: Mechanistic target of rapamycin complex 1
MODS: Multiple organ dysfunction syndrome
Nec-1: Necrostatin-1
NETs: Neutrophil extracellular traps
NF-κB: Nuclear factor-κB
NK: Natural killer
NLRs: Nucleotide-binding oligomerization domain-like receptors
NLRP3: NOD-like receptor family pyrin domain containing 3
NO: Nitric oxide
Nrf2: Nuclear factor E2-related factor 2
NSA: Necrosulfonamide
PAMPs: Pathogen-associated molecular patterns
PD-1: Programmed cell death protein 1
PICS: Persistent inflammation, immunosuppression, and catabolism syndrome
PLOOH: Phospholipid hydroperoxides
PMNs: Polymorphonuclear neutrophils
PPRs: Pattern recognition receptors
RHIM: RIP homotypic interaction motif
RIPK: Receptor-interacting protein kinase
RLRs: Retinoic acid-inducible gene I-like receptors
ROS: Reactive oxygen species
SA-AKI: Sepsis-associated acute kidney injury
SAE: Sepsis-associated encephalopathy
SIRS: Systemic inflammatory response syndrome
SIRT1: Sirtuin 1
SLC39A8: Solute carrier family 39 member 8
STAT: Signal transducers and activators of transcription
TFEB: Transcription factor EB
TGF-β1: Transforming growth factor β1
THBS1: Thrombospongoprotein 1
TLRs: Toll-like receptors
TNFR: Tumor necrosis factor receptor
TRADD: TNF receptor type 1-associated death domain protein
TRAILR: TNF-related apoptosis-inducing ligand receptor
Tregs: Regulatory T cells
ZBP1: Z-DNA-binding protein 1
